# Dexamethasone Selectively Inhibits Detachment of Metastatic Thyroid Cancer Cells during Random Positioning

**DOI:** 10.3390/cancers15061641

**Published:** 2023-03-07

**Authors:** Daniela Melnik, José Luis Cortés-Sánchez, Viviann Sandt, Stefan Kahlert, Sascha Kopp, Daniela Grimm, Marcus Krüger

**Affiliations:** 1 Department of Microgravity and Translational Regenerative Medicine, Otto von Guericke University, 39106 Magdeburg, Germany; 2 Research Group “Magdeburger Arbeitsgemeinschaft für Forschung unter Raumfahrt- und Schwerelosigkeitsbedingungen” (MARS), Otto von Guericke University, 39106 Magdeburg, Germany; 3 Institute of Anatomy, Otto von Guericke University, 39120 Magdeburg, Germany; 4 Core Facility Tissue Engineering, Otto von Guericke University, 39106 Magdeburg, Germany; 5 Department of Biomedicine, Aarhus University, 8000 Aarhus, Denmark

**Keywords:** glucocorticoids, thyroid cancer, random positioning, in vitro metastasis, cell adhesion, anti-adhesion, tight junctions, cancer treatment

## Abstract

**Simple Summary:**

Metastasis is the most dangerous feature of advanced cancers. In vitro models of this process could help us to study and understand mechanisms that are not readily accessible in the human body. In our approach, we used random positioning cell cultures to induce cancer cells to spread and form tumor spheroids. In this experimental setup, we show that dexamethasone is able to specifically inhibit the detachment of metastatic thyroid cancer cells. Our results not only show how differently healthy and malignant thyroid cells act and react in this in vitro metastasis model system, but also provide valuable insights into its functioning, possibilities and limitations.

**Abstract:**

We recently reported that synthetic glucocorticoid dexamethasone (DEX) is able to suppress metastasis-like spheroid formation in a culture of follicular thyroid cancer (FTC)-133 cells cultured under random positioning. We now show that this inhibition was selective for two metastatic thyroid carcinoma cells, FTC-133 and WRO, whereas benign Nthy-ori 3-1 thyrocytes and recurrent ML-1 follicular thyroid cancer cells were not affected by DEX. We then compare Nthy-ori 3-1 and FTC-133 cells concerning their adhesion and mechanosignaling. We demonstrate that DEX disrupts random positioning-triggered p38 stress signaling in FTC-133 cells, thereby antagonizing a variety of biological functions. Thus, DEX treatment of FTC-133 cells is associated with increased adhesiveness, which is mainly caused by the restored, pronounced formation of a normal number of tight junctions. Moreover, we show that Nthy-ori 3-1 and ML-1 cells upregulate the anti-adhesion protein mucin-1 during random positioning, presumably as a protection against mechanical stress. In summary, mechanical stress seems to be an important component in this metastasis model system that is processed differently by metastatic and healthy cells. The balance between adhesion, anti-adhesion and cell–cell connections enables detachment of adherent human cells on the random positioning machine—or not, allowing selective inhibition of thyroid in vitro metastasis by DEX.

## 1. Introduction

Cancer is considered one of the most life-threatening diseases due to its ability to metastasize. Metastasis is responsible for more than 90% of all cancer-related deaths [1]. The mechanism of cancer metastasis mainly involves six steps: (1) detachment from the primary tumor, (2) intravasation, (3) transport through the bloodstream, (4) entrapment in the endothelial walls of the distant organ, (5) extravasation into the parenchyma of the distant organ, and (6) formation of secondary tumors on the distant organ [2]. In most cases, the initialization of the metastatic process is thought to be the result of a genetic [3] or epigenetic [4] trigger. However, there is increasing evidence that mechanical stimuli also contribute to metastasis through the interplay of physical forces and biochemical signals (reviewed in [5,6]). In this context, it is important to note that not all cancer cells have the ability to form metastases. It is generally believed that a process similar to epithelial-mesenchymal transition (EMT) is required for cancer cells to metastasize [7], which is associated with a loss of adhesion and stiffness [8]. Early on, Guck et al. [9] demonstrated that human breast cancer cell lines MCF-7 and especially the highly metastatic MDA-MB-231 are more deformable than their epithelial counterpart MCF-10A and opened the field for cancer biophysics. That the invasiveness of cancer cells is indeed correlated with their plastic response was recently reconfirmed by Cho et al. [10] using a novel microfluidic system. Interestingly, metastatic cancer cells seem to change their mechanical properties in response to passive and active mechanical stimuli to a much greater extent than normal cells [11,12,13].

**Figure 1:**
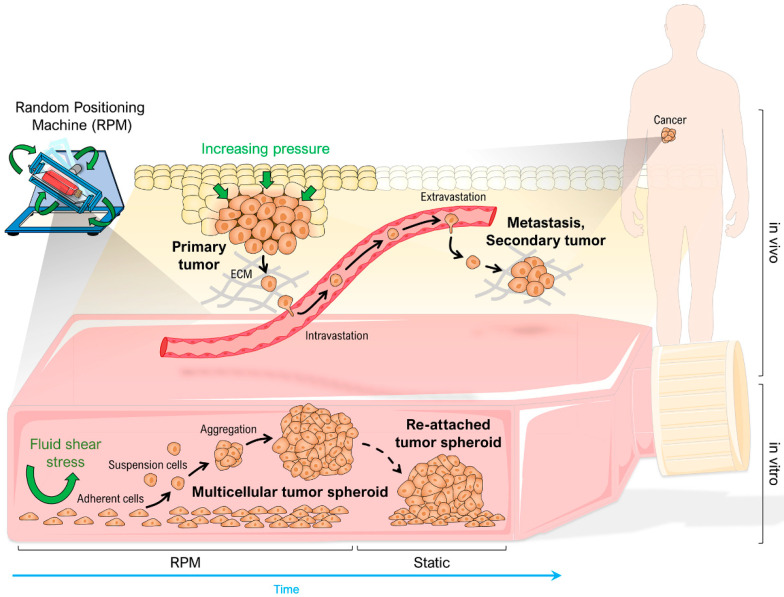
Comparison of metastasis in vivo and random positioning cell culture with adherent growing cancer cells in vitro. Modified from [19]. Parts of the figure were drawn by using pictures from Servier Medical Art, licensed under a Creative Commons Attribution 3.0 Unported License (https://creativecommons.org/licenses/by/3.0/, accessed on 12 February 2023).

The random positioning machine (RPM) is a rotating bioreactor originally developed for the study of gravitational biology in cells, tissues and organisms [14,15]. Unlike rotating clinostats, which are primarily designed to prevent sedimentation of suspension cells, the RPM generates shear forces as a side effect [16,17]. Computer simulations showed that the fluid motion inside the cell culture flasks on the RPM never reached or approached a steady state [18]. What is a disadvantage for some may be an advantage for others: adherent cells are stimulated by the RPM to detach from their surface, and in the case of tumor cells, this process mimics spreading in vivo in several aspects (Figure 1) [19,20,21]. Among the possible explanations for this in vitro metastasis model is that tumor cells respond differently and possibly more sensitively to mechanical stimuli. MDA-MB-231 cells with high metastatic potential were more susceptible to migration under fluid shear forces than the less metastatic MDA-MB-468. The benign MCF-10A cell line had the lowest migration potential under shear forces [22]. Fluid shear stress has also been described to promote invasion through increased secretion of matrix metalloproteinases (MMPs) [23], increased cell motility, and facilitated EMT of adherent tumor cells [24,25,26]. Ahn et al. [27] indicated that random positioning accelerates the migration of two types of adherent non-small cell lung cancer cells even in a floating environment. However, the exact mechanism of spheroid formation from 2D cultures on the RPM, which was also observed for several healthy cells [28,29,30], is still unclear.

Recently, we reported that RPM-induced spheroid formation of FTC-133 thyroid carcinoma cells was inhibited by dexamethasone (DEX) in a concentration-dependent manner and came across a complex regulation network of tumor spheroid formation under dynamic flow that may be influenced by glucocorticoids such as DEX [31]. With the current study, we aimed to reveal and explain the effects of DEX treatment on thyroid cancer cells cultured on the RPM and generally gain a better understanding of the behavior of human cells in a rotational culture.

## 2. Materials and Methods

### 2.1. Cell Lines and Cell Culture

The human follicular thyroid carcinoma cell lines FTC-133 (passages 17–25) and WRO (passages 10–16) and the noncancerous follicular epithelial cell line Nthy-ori 3-1 (passages 10–15) were purchased from Sigma-Aldrich (St. Louis, MO, USA). The human follicular thyroid carcinoma cell line ML-1 (passages 5–9) isolated from recurrence [32] was used from a laboratory stock. Cells were cultured in RPMI 1640 medium (Life Technologies, Carlsbad, CA, USA) supplemented with 10% fetal calf serum (FCS; Sigma-Aldrich, St. Louis, MO, USA), and 1% penicillin/streptomycin (Life Technologies) at 37 °C and 5% CO_2_ until used for experiments. Twenty-four hours before each experiment, a cell density of 1 × 10^6^ cells per flask were seeded in T25 cell culture flasks (Sarstedt, Nümbrecht, Germany) to allow cells to adhere. T25 flasks equipped with glass coverslips and a reduced cell density of 0.5 × 10^6^ cells were used for immunofluorescence staining.

Breast epithelial cells (MCF-10A; ATCC, Manassas, VA, USA), mammary carcinoma cells (MCF-7; ATCC), and prostate carcinoma cells (PC-3, LnCAP; ATCC) for supplemental experiments were cultured accordingly. The MCF-10A cell line was cultured in DMEM/F12 medium supplemented with 0.5% Mammary Epithelial Growth Supplement (MEGS) (Life Technologies).

### 2.2. Dexamethasone and Drug Treatment

Ethanol-soluble DEX was purchased from Sigma-Aldrich. Twenty-four hours after seeding, cells were washed once with phosphate-buffered saline (PBS; Life Technologies), synchronized for four hours in RPMI 1640 with 0.25% FCS and 1% Penicillin/Streptomycin, following a cultivation with RPMI 1640 medium supplemented with 0–1000 nM DEX, as described in [31]. This procedure was applied before any drug treatment.

To investigate the selective effect of DEX on metastatic cell lines, we treated FTC-133 and WRO cells with the re-activator of mutant p53 activity, PRIMA-1^Met^ (2-(Hydroxymethyl)-2-(methoxymethyl)-1-azabicyclo [2.2.2]octan-3-one; Sigma-Aldrich). PRIMA-1^Met^ was dissolved in water. Cell cultures were supplemented with 10 µM PRIMA-1^Met^ or 10 µM PRIMA-1^Met^ combined with 1 µM DEX for three days.

To target GR signaling, we used the water-soluble GR antagonist Mifepristone (MIF, Sigma-Aldrich). Based on the highest DEX concentration of 1 µM, FTC-133 cells were cultured for three days in DEX:MIF ratios of 1:1, 1:10 and 1:20. Untreated cells, as well as cells treated with 1 µM DEX or 20 µM MIF, served as controls.

Moreover, we applied the D-amino acid cell-penetrating peptide inhibitor of MUC-1, GO-203 (Selleck Chemicals, Planegg, Germany). A GO-203 stock solution was prepared with sterile water. According to the manufacturer’s instructions, we treated Nthy-ori 3-1 cells with 5 µM of GO-203 in combination with 1 µM DEX (Sigma-Aldrich) for three days. To exclude side effects of GO-203, controls supplemented with GO-203 and untreated controls were included.

### 2.3. Random Positioning

Cell cultures were randomly positioned on a desktop RPM (Dutch Space, Leiden, The Netherlands) inside an incubator or inside an incubator RPM (iRPM 2.0, designed and constructed by Prof. Jörg Sekler, University of Applied Sciences and Arts Northwestern Switzerland [33]), both operated in a real random mode. Before the run, all culture flasks were filled bubble-free with medium supplemented with the corresponding drug concentrations. Corresponding static controls were each prepared in parallel under the same conditions and stored next to the device in an incubator.

### 2.4. Static Forced Floating Spheroid Formation

Cells were seeded in 96-well BIOFLOAT™ U-bottom plates (faCellitate, Mannheim, Germany) at a density of 2500 cells per well with culture medium supplemented with 0, 10, 100 and 1000 nM DEX at 37 °C and 5% CO_2_. Images were acquired every 24 h for three days using an Invitrogen EVOS^TM^ XL Core Imaging System (Thermo Fisher Scientific, Waltham, MA, USA).

### 2.5. Trypsin Digestion Adhesion Assay

To investigate the effect of DEX and random positioning on the “stickiness” of adherent FTC-133 and Nthy-ori 3-1 cells, we seeded 0.5 × 10^6^ cells in a Slide Flask (Thermo Fisher Scientific). Cells were treated as described in Section 2.2. After a 24-h attachment period, the Slide Flasks were completely filled with RPMI 1640 medium supplemented with 1 µM DEX for the DEX treatment group and cultured for 24 h under static or RPM conditions. After 24 h, cells were photographed, washed once with PBS, and incubated for 9 min (Nthy-ori 3-1) or 5 min (FTC-133) in prewarmed 0.05% trypsin-EDTA (Life Technologies) at room temperature (RT). The Slide Flasks were moderately hit once, fresh medium was added and removed to stop the trypsin reaction, and the remaining cells were imaged under the microscope.

### 2.6. Phase Contrast Microscopy

Cells were observed and photographed using an OLYMPUS CKX53 inverted microscope (Olympus, Tokio, Japan) or an Invitrogen^TM^ EVOS^TM^ XL Core Imaging System (Thermo Fisher Scientific).

### 2.7. Immunofluorescence Microscopy

The adherent cells were washed twice with PBS and fixed by incubation for 30 min at 4 °C in 100% EtOH (Carl Roth, Karlsruhe, Germany), followed by 90 s in ice-cold acetone (Carl Roth) at RT. Cells were stored in 0.5 M sodium azide (Carl Roth) in 0.1 M PBS at 4 °C until observation. Fixed cells were blocked with 3% bovine serum albumin (BSA, Carl Roth) in 0.1 M PBS for 1 h at RT. Subsequently, the cells were labeled with the primary antibodies diluted in 1% BSA (listed in Table S1) overnight at 4 °C. The next day, the cells were washed three times with 0.1 M PBS and incubated with the secondary Alexa Fluor™ 488 (AF488)-conjugated anti-rabbit (Invitrogen, Life Technologies) or Alexa Fluor™ 647 (AF647)-conjugated anti-mouse (Invitrogen, Life Technologies) antibodies at a dilution of 1:500 or 1:1000 at RT for 1 h. Cells were washed again three times with 0.1 M PBS and mounted with Fluoroshield^TM^ with DAPI (4′,6-diamidino-2-phenylindole) (Sigma-Aldrich). The next day, the slides were examined using a ZEISS LSM 800 confocal laser scanning microscope (Carl Zeiss, Oberkochen, Germany). To ensure comparability for intensity quantification, all images were acquired with the same settings using the ZEISS Airyscan detector and ZEN 3.4 software (Carl Zeiss). Images were normalized to the untreated FTC-133 samples cultured under static conditions. Airyscan processing settings were optimized for each antibody/wavelength combination and manually applied to corresponding samples. The resulting file was used in Fiji software v1.53t (ImageJ, imagej.net) to quantify the relative protein amount based on the fluorescence of the sample with the “Image Calculator” tool. Relative intensities were measured according to the method recently described by Shihan et al. [34]. To determine the ratio of protein localization between the nucleus and cytoplasm, the fluorescence intensities of the protein of interest were measured for the nuclear area and the cytoplasm. The nuclear area was determined before using the DAPI signal. For the co-localization measurement of assembled protein complexes, ImageJ image calculation was used to combine the channels of interest and to subtract the DAPI channel. The amount of protein complexes/co-localization was indicated by the LUT fire setting.

### 2.8. mRNA Isolation and Quantitative Real-Time PCR

The adherent cells were washed once with PBS and fixed by addition of Invitrogen RNA*later*^TM^ solution (Life Technologies). Cells were harvested using cell scrapers and samples were stored at −20 °C until mRNA extraction. mRNA isolation was performed by using the RNeasy Mini Kit (Qiagen, Venlo, The Netherlands), following the manufacturer’s protocol. RNA concentration was determined with a NanoPhotometer^®^ N60 (Implen, Munich, Germany) followed by cDNA synthesis using the High Capacity cDNA Reverse Transcription Kit (Life Technologies). To define the expression level of the genes of interest, quantitative real-time PCR was performed by using the Fast SYBR™ Green Master Mix (Life Technologies) and the QuantStudio™ 3 Real-Time PCR System (Thermo Fisher Scientific). Primer sequences used in the quantitative real-time PCR can be found in the Supplementary Materials (Table S2). The samples were measured in triplicates and evaluated by using the comparative threshold cycle (ΔΔC_T_) method with 18S rRNA as the housekeeper reference.

### 2.9. Protein Isolation and Western Blot Analysis

The adherent cells were placed on ice, washed once with ice-cold PBS, and then harvested with ice-cold PBS using cell scrapers. The suspensions were centrifuged at 3000× *g* for 10 min at 4 °C, then the PBS was discarded and the cell pellets were stored at −150 °C.

Proteins were isolated by performing incubation and centrifugation steps in RIPA lysis buffer (Sigma-Aldrich). Protein concentration was measured using the BCA protein assay (Carl Roth). First, 20 µg total protein were loaded onto an SDS-PAGE gel (8%, 10% or 12.5%, 1 mm), run for 20–30 min at 80 V, 90 min at 100 V, and transferred to a nitrocellulose membrane (Carl Roth; 90 min, 100 V). Membranes were blocked with 5% BSA (Carl Roth) in TBS-T for 1 h at RT. To detect proteins of interest, membranes were incubated overnight at 4 °C with a primary antibody diluted in TBS-T containing 5% BSA. GAPDH was used as a loading control. Afterwards, the membranes were washed three times with TBS-T for 5 min and incubated for 1 h at RT with a horseradish peroxidase (HRP)-linked secondary antibody (Invitrogen, Life Technologies) diluted in TBS-T containing 5% BSA. Before the protein bands were visualized by applying Clarity Western ECL Substrate (Bio-Rad, Hercules, CA, USA), the membrane was washed three times with TBS for 5 min. Images were acquired using the ChemiDoc^TM^ XRS+ molecular imager (Bio-Rad) and analyzed using Image Lab^TM^ 6.1 software (Bio-Rad).

For re-staining (e.g., to visualize loading controls of similar size to the protein of interest), membranes were stripped in some Western blots. To remove antibodies, membranes were incubated in stripping buffer (62.5 mM TRIS, 2% SDS, pH 6.8) at 50 °C for 15–20 min and then washed three times with TBS for 5 min.

All antibodies used for Western blot analyses can be found in the Supplementary Materials (Table S1).

### 2.10. Cell Culture Supernatant Analyses

Oxygen concentration was measured directly in the T25 cell culture flask by fiber-optic oxygen detection using a Microx TX3 transmitter equipped with a flat, broken-tip oxygen microsensor (PreSens, Regensburg, Germany). The sensor was calibrated according to the manufacturer’s specifications. Temperature-compensated measurements (37 °C) were recorded using TX3v602 software (PreSens) over a period of 5 min.

### 2.11. Spheroid Measurement and Quantification

Spheroids were generated through the cultivation of adherent or suspended cells on the RPM. Images of spheroids were taken by using the microscopes described in Section 2.10. These were randomly selected and imaged with the 4× lens. The analysis was done with ImageJ. First, the spheroids of five images were counted, then outlined with the ImageJ tool “freehand selections” and automatically measured.

### 2.12. Statistics

Statistical evaluation was performed using SPSS Statistics v28 (IBM, Armonk, NY, USA). The nonparametric Mann–Whitney U test or the independent *t*-test (for Western blot and O_2_ saturation analyses due to the smaller number of samples) was used to compare samples from different culture conditions. All data are presented as mean ± standard deviation (SD) with a significance level of *p* < 0.05.

## 3. Results

**Figure 2:**
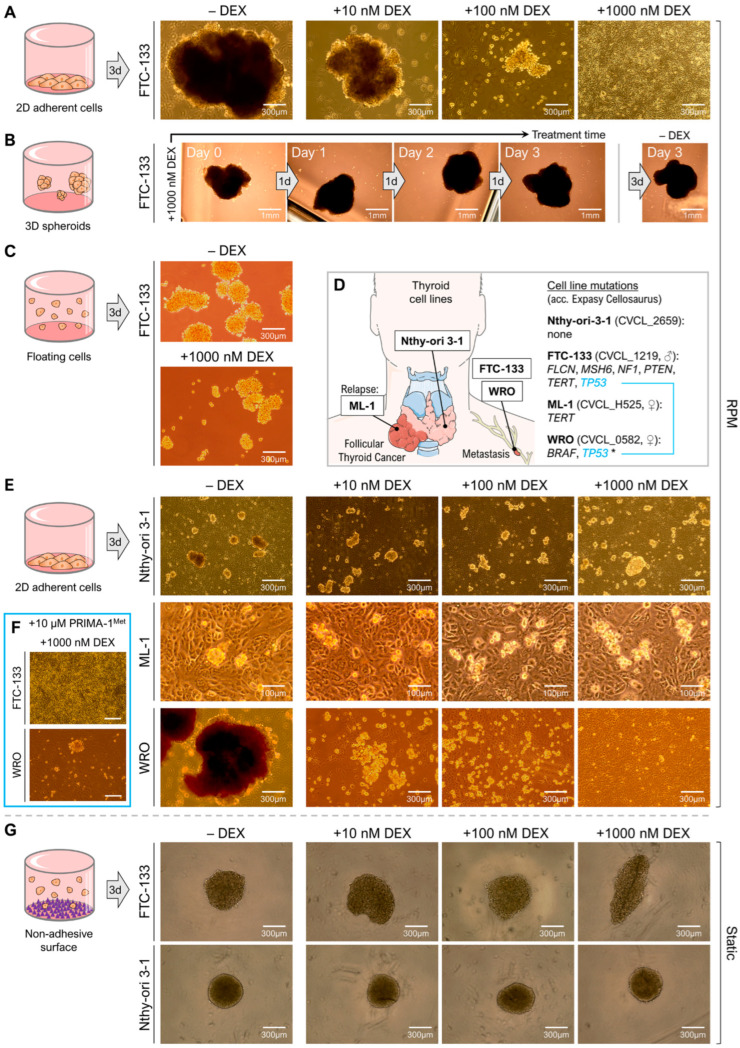
Impact of different DEX concentrations (0, 10, 100, 1000 nM) on spheroid formation and stability of different thyroid epithelial cells after 3 days. (**A**) Spheroid formation of adherent FTC-133 metastasized carcinoma cells after rotation on the RPM; (**B**) stability of preformed FTC-133 spheroids on the RPM in the presence of 1000 nM DEX; (**C**) spheroid formation of FTC-133 suspension cells; (**D**) origins of the used thyroid cell lines and documented cell line mutations (https://www.cellosaurus.org; accessed on: 2 February 2023). ♂, ♀ indicate the gender of the donor. * The *TP53 P223L* mutation in WRO cells is only reported by some authors; (**E**) spheroid formation of adherent Nthy-ori 3-1 thyroid epithelial, ML-1 recurrent and WRO metastasized thyroid carcinoma cells after 3d rotation on the RPM; (**F**) spheroid formation of FTC-133 and WRO cells in the presence of 10 µM of the p53 reactivator PRIMA-1^Met^ and 1000 nM DEX; (**G**) spheroid formation of FTC-133 and Nthy-ori 3-1 cells under static forced floating conditions in ultra-low attachment plates. Parts of subfigure D were drawn by using pictures from Servier Medical Art, licensed under a Creative Commons Attribution 3.0 Unported License (https://creativecommons.org/licenses/by/3.0/, accessed on 12 February 2023).

As previously reported, FTC-133 spheroid formation was inhibited by DEX in a concentration-dependent manner (Figure 2A).

### 3.1. Dexamethasone Inhibits Formation but Not Stability of Multicellular Tumor Spheroids

To test spheroid integrity during DEX treatment, we added 1000 nM DEX to a culture of FTC-133 spheroids formed after three days on the RPM and continued random positioning. After three more days, we could see that the spheroids had not disassembled or shrunk, but continued growing in the presence of DEX (Figure 2B). DEX had no influence on the stability of preformed tumor spheroids on the RPM.

### 3.2. Dexamethasone Selectively Affects Spheroid Formation of Metastatic Thyroid Cancer Cells

In addition to FTC-133 cells, we tested spheroid formation of the normal Nthy-ori 3-1 thyroid epithelial cell line and recurrent ML-1 carcinoma cells derived from a dedifferentiated follicular thyroid carcinoma relapse (Figure 2D) in the presence of different DEX concentrations (0, 10, 100, 1000 nM). Both cell lines formed multicellular spheroids after 72 h on the RPM (Figure 2E). Although the Nthy-ori 3-1 and ML-1 spheroids were much smaller compared to the FTC-133 spheroids, formation was not inhibited by increasing DEX concentrations (Figure 2E). Furthermore, we tested a second metastasis-born thyroid cell line, WRO, that also showed a highly reduced formation of 3D aggregates in the presence of DEX (Figure 2E, bottom line).

The results suggested a selective effect of DEX on the RPM-triggered 3D growth of thyroid carcinoma cells isolated from metastases. Because both FTC-133 and WRO metastatic cell lines have *TP53* mutations, PRIMA-1^Met^ (APR-246) was used to restore mutant p53 activity, thus eliminating the possibility that the observed effect of DEX was due to p53 dysfunction. We demonstrated that the addition of 10 µM PRIMA-1^Met^ did not affect the DEX inhibition of spheroid formation in FTC-133 cells and could not completely reverse the effect in WRO cells (Figure 2F), ruling out the involvement of p53 in this process. Furthermore, PRIMA-1^Met^ alone had no effect on spheroid formation on the RPM.

### 3.3. Dexamethasone Supresses RPM-Induced Cell Detachment but Not 3D Growth in General

RPM-induced spheroid formation comprises two steps: The detachment of the actually adherent cells from their growth surface and the aggregation/growth of the detached and floating cells in the medium. To find out which step is influenced by DEX, we used ultra-low attachment multi-well plates for comparison, in which spheroids form automatically without mechanical action. After 72 h, spheroids of FTC-133 cells and Nthy-ori 3-1 cells have formed in the wells independently of the used DEX concentration (0–1000 nM) (Figure 2G). Furthermore, FTC-133 cells formed smaller spheroids in the presence of 1000 nM DEX when they were placed directly on the RPM without a prior attachment phase (Figure 2C). The number of these spheroids was comparable with those of the DEX-free samples. We also observed 3D growth when we cultured FTC-133 suspension cells together with DEX on a clinostat. Accordingly, DEX did not prevent the aggregation of the cells itself, but seems to impede the ‘metastasis-like’ detachment of thyroid cancer cells observed under random positioning.

### 3.4. Dexamethasone Treatment Alters ‘Stickiness’ of the Cells

**Figure 3:**
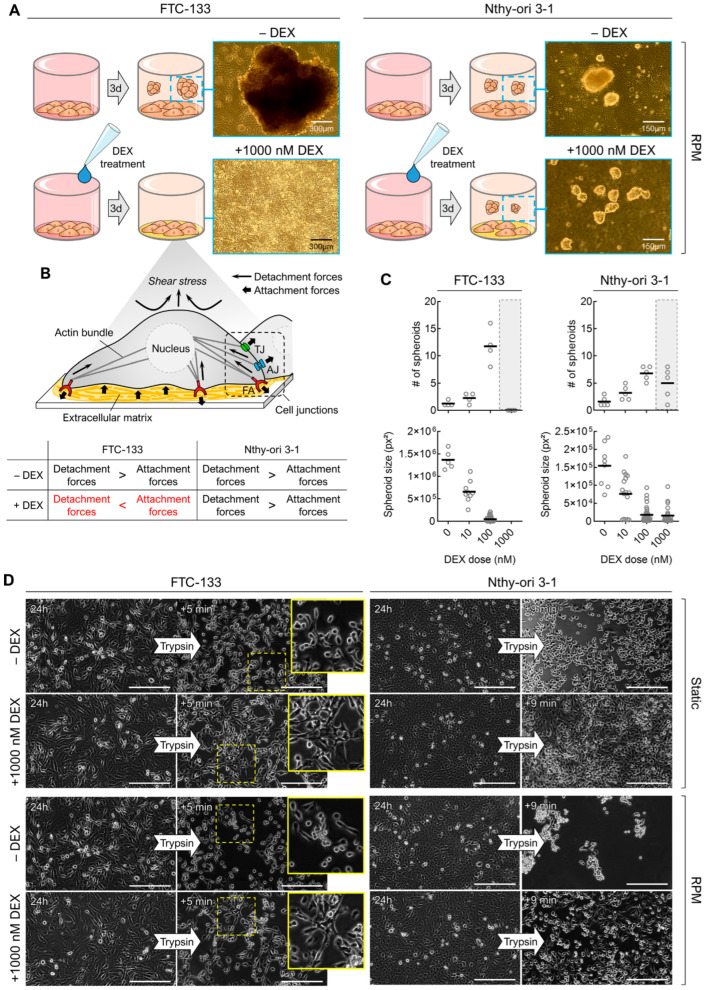
(**A**) Observed effects of 1000 nM DEX on the RPM-induced spheroid formation of FTC-133 und Nthy-ori 3-1 cells after 3 days; (**B**) putative reasons for the different behavior of the two cell lines. AJ: adherens junction, FA: focal adhesion, TJ: tight junction; (**C**) number and size of spheroids formed after 3 days on the RPM depending on DEX concentration; (**D**) trypsin-based cell detachment assay reveals the general adhesiveness of cells after 24 h of culture in the presence and absence of DEX. The yellow boxes show an enlargement of the outlined section. Scale bars: 300 µm.

After showing specifically that the spheroid formation of FTC-133 cancer cells versus benign Nthy-ori 3-1 cells is affected by DEX (Figure 3A), we started to investigate the cause in these two cell lines. Considering the different forces of cell adhesion and shear forces on RPM (Figure 3B), DEX treatment of FTC-133 cells must result in adhesion forces being greater than shear forces leading to cell detachment. When treated with a DEX concentration of 1000 nM, spheroid formation of FTC-133 cells was completely suppressed in contrast to Nthy-ori 3-1 cells (Figure 3C, grey area). Spheroid size was also affected by DEX, but since the focus of this study was on the formation of spheroids rather than their later development, this aspect was not further explored here. A trypsinization assay showed that DEX increased the adhesiveness of both cell lines, and that random positioning deceased the adhesiveness of Nthy-ori 3-1 cells.

FTC-133 cells showed a more bipolar or multipolar shape with cellular bridging in the presence of DEX (Figure 3D, yellow arrows). Factors that may increase cell adhesion include extracellular matrix composition (ECM) proteins and cell–cell or cell–ECM junctions.

#### 3.4.1. Extracellular Matrix

**Figure 4:**
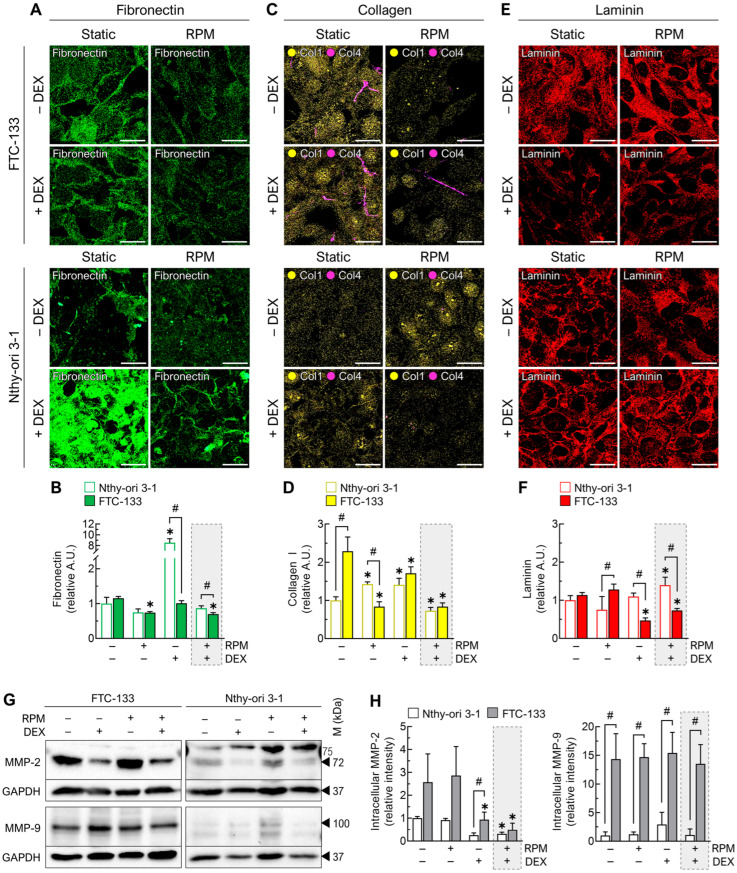
Effect of DEX (1000 nM) and random positioning on the ECM of FTC-133 and Nthy-ori 3-1 cells after 3 days. (**A**) Immunofluorescence of fibronectin; (**B**) fluorescence-based quantification of fibronectin under the different culture conditions (*n* = 5); (**C**) immunofluorescence of collagen I (yellow) and collagen IV (purple); (**D**) fluorescence-based quantification of collagen I under the different culture conditions (*n* = 5); (**E**) immunofluorescence of laminin; (**F**) fluorescence-based quantification of laminin under the different culture conditions (*n* = 5); (**G**) intracellular protein levels of matrix metallopeptidases MMP-2 and MMP-9 indicated by Western blot (see also Supplementary File S1); (**H**) densitometric quantification of Western blot proteins (*n* = 3). Scale bars: 300 µm. * *p* < 0.05 vs. control condition (−DEX/−RPM); ^#^ *p* < 0.05.

We examined the ECM composition based on the immunofluorescence of several major components, including fibronectin, collagen I/IV and laminin. Whereas DEX had no effect on fibronectin in FTC-133 cells, we found an 8-fold increase of fibronectin in DEX-treated Nthy-ori 3-1 cells. Moreover, we detected a decrease in fibronectin in RPM-grown FTC-133 cells, which was also present, although to a lesser extent, in Nthy-ori 3-1 cells (Figure 4A,B). In the RPM+DEX samples, the FTC-133 cells produced a slightly lower amount of fibronectin than the Nthy-ori 3-1 cells (Figure 4B).

Collagen I was more highly expressed in FTC-133 cells. Random positioning and DEX treatment alone increased collagen I levels in Nthy-ori 3-1 cells and decreased them simultaneously in FTC-133 (Figure 4C,D). Thus, collagen I levels of both cell lines were almost identical after 72 h of DEX treatment. Interestingly, DEX treatment during random positioning lead to a decrease in collagen I in both malignant and healthy cells (Figure 4D). Collagen IV was only detectable in significant amounts in FTC-133 cells after 72 h. It was less present after random positioning (Figure 4C).

Laminin was significantly reduced by DEX in FTC-133 cells, regardless of culture condition (Figure 4E,F). In Nthy-ori 3-1 cells there was a slight increase of laminin in the DEX-treated RPM group (Figure 4F).

While the amount of MMP-9 was not affected by either RPM or DEX, MMP-2 was decreased after DEX administration in both FTC-133 and Nthy-ori 3-1 cells. Nthy-ori 3-1 cells exhibited generally lower levels of MMP-2 and MMP-9. In particular, MMP-9 was increased 14-fold in FTC-133 cells (Figure 4G,H).

In summary, we showed that, in FTC-133 cells, three major ECM structural components were reduced by DEX treatment during random positioning. Compared with Nthy-ori 3-1 cells, the ECM components were even more reduced. In addition, FTC-133 cells produced many more collagenases. All this cannot explain the different response of the two cell lines to DEX treatment.

#### 3.4.2. Cell Junctions

**Figure 5:**
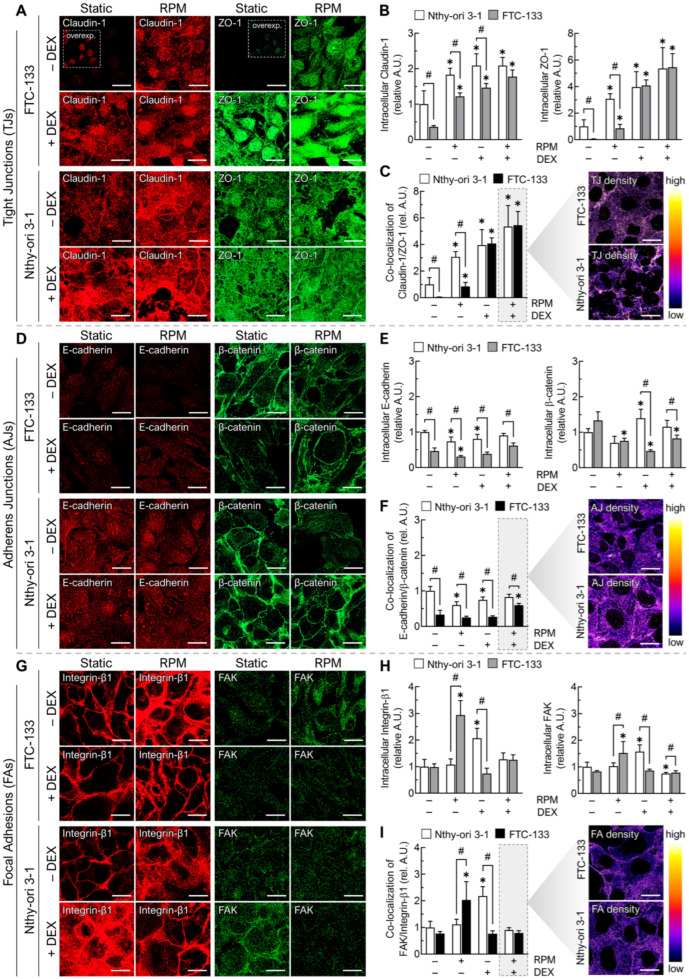
Effect of DEX (1000 nM) and random positioning on the cell junctions of FTC-133 and Nthy-ori 3-1 cells after 3 days. (**A**) Immunofluorescence of claudin-1 (red) and ZO-1 (green). The small inserts show low signal pictures with higher brightness; (**B**) fluorescence-based quantification of claudin-1 and ZO-1 under the different culture conditions (*n* = 5); (**C**) co-localization of claudin-1 and ZO-1; (**D**) immunofluorescence of E-cadherin (red) and β-catenin (green); (**E**) fluorescence-based quantification of E-cadherin and β-catenin under the different culture conditions (*n* = 5); (**F**) co-localization of E-cadherin and β-catenin; (**G**) immunofluorescence of integrin-β1 (red) and FAK (green); (**H**) fluorescence-based quantification of integrin-β1 and FAK under the different culture conditions (*n* = 5); (**I**) co-localization of integrin-β1 and FAK. Scale bars: 300 µm. * *p* < 0.05 vs. control condition (−DEX/−RPM); ^#^ *p* < 0.05.

The expression of the tight junction (TJ) proteins claudin-1 and ZO-1 (zonula occludens 1) was significantly lower in FTC-133 cells than in Nthy-ori 3-1 cells under normal culture conditions. However, in both cell lines, the levels of the proteins were increased by both DEX and, to a lesser extent, by the RPM (Figure 5A,B). In particular, the expression of ZO-1 was increased 27-fold in FTC-133 in the presence of DEX and 20-fold after exposure to the RPM. In addition, FTC-133 cells showed increased nuclear localization of ZO-1 both on the RPM and after DEX treatment (Figure 5A), indicating the formation and maturation of TJs [35].

Co-localization analysis of claudin-1 and ZO-1 confirmed the enhanced formation of TJs by DEX, which reached the same amount in FTC-133 cells as in Nthy-ori 3-1 cells after 72 h, regardless of culture motion (Figure 5C). This increase in TJs could also explain the cellular bridging and altered shape of FTC-133 cells in the presence of DEX (Figure 3D). Another zonula occludens protein, ZO-2, showed similar regulation to ZO-1 in FTC-133 cells (Figure S1A). Overall, both FTC-133 cells and WRO cells showed very low baseline expression of claudin-1 under static cell culture and strong upregulation during DEX treatment on the RPM (Figure S1B). Therefore, TJ regulation might be related to the inhibition of spheroid formation of both cell lines by DEX (Figure S1C).

E-cadherin expression was generally lower in FTC-133 cells than in Nthy-ori 3-1 cells. Whereas E-cadherin was downregulated mainly by RPM in both cell lines, in FTC-133 cells, both DEX and RPM had a suppressive effect on β-catenin levels (Figure 5D,E). Overall, the amount of adherens junctions (AJs) was higher in FTC-133 cells in the RPM+DEX samples than in standard culture, but still significantly lower than in Nthy-ori 3-1 cells (Figure 5F).

Unlike the benign cells, there was an increase in focal adhesions (FAs) in FTC-133 cancer cells in response to the RPM (Figure 5G–I). In Nthy-ori 3-1 cells, a similar effect was detected in response to DEX. This is also reflected in the amount of the single components, FAK (focal adhesion kinase) and integrin-β1 (Figure 5H). Nevertheless, the combination RPM+DEX did not show any changes in the number of FAs in either cell line (Figure 5I).

In summary, although FTC-133 cells showed a significant increase in claudin-based TJs and cadherin-based AJs after DEX treatment on RPM, the number of cellular junctions examined in these experiments was not higher than that in Nthy-ori 3-1 cells.

#### 3.4.3. Anti-Adhesion Molecules

Because neither the ECM composition nor the number of cellular junctions could explain the different response of the two cell lines to DEX treatment, we further investigated anti-adhesion effects. Thus, we examined the anti-adhesion molecule mucin-1 (MUC1), which is known to inhibit cell–cell and cell–ECM interactions [36,37].

**Figure 6:**
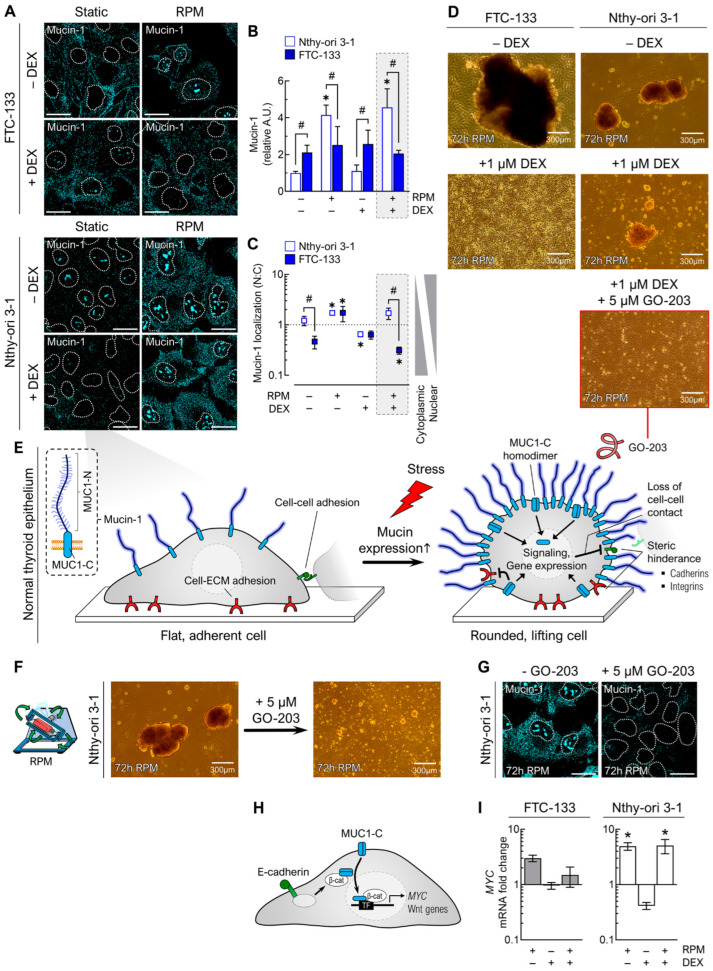
Effect of DEX (1 µM) and random positioning on mucin-1 in FTC-133 and Nthy-ori 3-1 cells after 3 days. (**A**) Mucin-1 immunofluorescence in adherently growing cells. Mucin-1 is shown in turquoise, with the outlines of the nuclei as dashed lines; (**B**) fluorescence-based quantification of mucin-1 under the different culture conditions (*n* = 5); (**C**) nuclear localization of mucin-1 (*n* = 5); (**D**) spheroid formation of FTC-133 and Nthy-ori 3-1 cells in the presence of 1 µM DEX and 5 µM of the MUC1-inhibitor GO-203; (**E**) hypothetical model that could explain the anti-adhesion of Nthy-ori 3-1 cells during RPM-induced increased mucin-1 expression. Mucin-1 is a transmembrane glycoprotein consisting of two subunits, an extracellular, N-terminal highly glycosylated subunit (MUC1-N) and a C-terminal transmembrane subunit (MUC1-C). MUC1-C can form homodimers that affect cellular signaling pathways and gene expression; (**F**) spheroid formation of Nthy-ori 3-1 cells in the presence of 5 µM GO-203; (**G**) MUC-1 immunofluorescence (turquoise) of adherently growing Nthy-ori 3-1 cells in absence and presence of 5 µM GO-203. (**H**) MUC1-C activates the Wnt/β-catenin pathway and induces *MYC* expression. TF: transcription factor. (**I**) Expression levels (mRNA) of the *MYC* gene (*n* = 5). Scale bars: 300 µm. * *p* < 0.05 vs. control condition (−DEX/−RPM); ^#^ *p* < 0.05.

According to the literature [38], the oncoprotein mucin-1 was more highly expressed in FTC-133 cells than in Nthy-ori 3-1 cells under static culture conditions. In FTC-133 cells, neither random positioning nor DEX treatment had any effect on mucin-1 levels (Figure 6A,B). However, immunofluorescence revealed a significant increase of mucin-1 in Nthy-ori 3-1 cells cultured on the RPM (Figure 6A,B). We also confirmed this effect for ML-1 cells (similar response to DEX as Nthy-ori 3-1), but not for WRO cells (similar response to DEX as FTC-133) (Figure S2A). Treatment with 5 µM of the MUC1-inhibitor GO-203 showed that DEX could also suppress RPM-induced spheroid formation of Nthy-ori 3-1 cells after inhibition of mucin-1 (Figure 6D). In addition, we found an attenuated ability for spheroid formation when we cultured Nthy-ori 3-1 cells in the presence of GO-203 on the RPM (Figure 6E,F). Moreover, the amount of mucin-1 in the presence of GO-203 was decreased after 72 h on the RPM (Figure 6G).

The C-terminal transmembrane subunit of mucin-1 (MUC1-C) can activate the Wnt/β-catenin pathway and induce expression of *MYC* and other Wnt genes (Figure 6H). Consistent with the immunofluorescence data of Nthy-ori 3-1 cells (Figure 6A–C), the expression of *MYC* was significantly increased on the RPM (Figure 6I).

### 3.5. Dexamethasone Influences Signal Transduction Pathways

#### 3.5.1. Glucocorticoid Receptor Signaling

**Figure 7:**
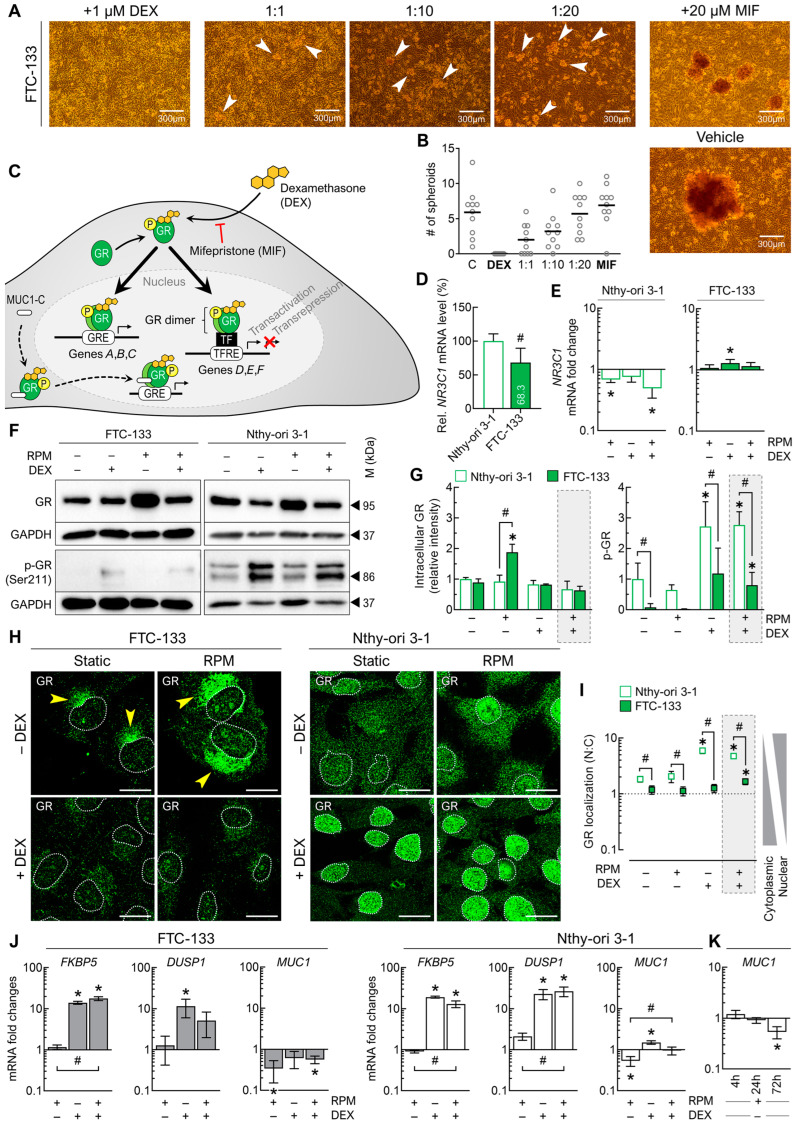
Effect of DEX on the glucocorticoid receptor (GR) and GR signaling in FTC-133 and Nthy-ori 3-1 cells after 3 days. (**A**) Spheroid formation (white arrows) of FTC-133 cells in the presence of 1 µM DEX and different concentrations (1–20 µM) of the competitive GR inhibitor mifepristone (MIF); (**B**) number of FTC-133 spheroids formed on the RPM in the presence of DEX and MIF (*n* = 10). (**C**) Simplified schematic representation GR’s mechanism of action. The genomic effects of DEX occur through its binding to GR and the movement of DEX-GR to the nucleus, where they can regulate the transcription of genes. GRE: glucocorticoid response element, TF: transcription factor, TFRE: transcription factor response element. (**D**) Expression levels (mRNA) of the *NR3C1* gene (*n* = 5); (**E**) fold changes of *NR3C1* expression under the different culture conditions (*n* = 5); (**F**) intracellular protein levels of GR and phosphorylated GR (p-GR) indicated by Western blot (see also Supplementary File S1); (**G**) densitometric quantification of Western blot proteins (*n* = 3); (**H**) GR immunofluorescence in adherently growing cells. GR is shown in green, with the outlines of the nuclei as dashed lines. In DEX-free FTC-133 cultures, GR is located near the nucleus (yellow arrows). (**I**) Nuclear localization of GR (*n* = 5); (**J**) fold changes in the expression of some signature genes of the GR pathway under the different culture conditions (*n* = 5); (**K**) fold changes in *MUC1* expression on the RPM after different time points (*n* = 5). Scale bars: 300 µm. * *p* < 0.05 vs. control condition (−DEX/−RPM); ^#^ *p* < 0.05.

The main actions of DEX occur through the activation of the glucocorticoid receptor (GR, gene symbol: *NR3C1*), which is activated by phosphorylation and can actively shuttle between cytoplasm and nucleus, where it acts as a transcription factor (Figure 7C) [39]. To verify whether GR effects were responsible for the observed spheroid suppression in FTC-133 cells, we used the competitive GR inhibitor mifepristone (MIF). We found that increasing amounts of MIF (1–20 µM) in the presence of DEX (1 µM) indeed facilitated the (re-)formation of small tumor spheroids.

However, the spheroids in the presence of MIF were smaller than in the vehicle samples (Figure 7A), maybe due to its known anti-proliferative effect. Quantification of the spheroids showed very well the competitive principle of MIF and DEX: with increasing MIF concentration, the number of spheroids formed approaches of the untreated control group (Figure 7B).

With this information, we took a closer look at GR activity under random positioning and DEX treatment. Under static cell culture conditions, expression of the *NR3C1* gene was lower in FTC-133 cells (68.3%) compared to Nthy-ori 3-1 (Figure 7D). Moreover, *NR3C1* transcription in the two cell lines was affected differently by external conditions: While *NR3C1* mRNA levels in FTC-133 cells were increased by DEX, they were decreased in Nthy-ori 3-1 cells by random positioning (Figure 7E).

Western blot showed an increased GR amount in FTC-133 cells on the RPM in the absence of DEX. We also observed a generally lower amount of “activated” p-GR (Ser211) in Nthy-ori 3-1 cells and a strong increase in p-GR after the addition of DEX in both cell lines (Figure 7F,G).

In the absence of DEX, GR was mainly located in the cytoplasm near the nucleus of FTC-133 cells (Figure 7H, yellow arrows). In the presence of DEX, the amount of GR was reduced, but little was translocated to the nucleus (Figure 7H,I). GR translocation was not significantly altered by random positioning after 72 h, suggesting that the RPM has no major influence on normal GR behavior in FTCs. In Nthy-ori 3-1 cells, GR was significantly translocated to the nucleus during DEX treatment (Figure 7H,I).

At the gene expression level, the GR target genes *FKBP5* and *DUSP1* were found to be upregulated in both cell lines in the presence of DEX under both rotational and static conditions (Figure 7J).

However, the upregulation of *DUSP1* (encoding dual specificity protein phosphatase 1) in FTC-133 cells on the RPM was lower than in the static cell culture. In addition, one other mutual target gene of GR, *MUC1* (encoding mucin-1)*,* was downregulated in FTC-133 cells primarily by the RPM, whereas in Nthy-ori 3-1 cells it was decreased by random positioning but also upregulated by DEX (Figure 7J). The observed overexpression of mucin-1 protein in Nthy-ori 3-1 cells on the RPM (Figure 6A) could be explained by a transient upregulation of *MUC1* and subsequent negative feedback after 72 h (Figure 7K).

Since a large percentage of GR binding sites is known to be hypoxia-specific [40,41,42] and we needed to work with completely filled cell culture flasks for random positioning, we also checked whether there was a gene expression response to hypoxia via hypoxia-inducible factor 1α (HIF1A). We found that *HIF1A* expression was upregulated only in Nthy-ori 3-1 cells when the culture flasks were completely filled (Figure S3). This change was independent of RPM exposure or DEX treatment.

#### 3.5.2. Cell Stress Signaling

**Figure 8:**
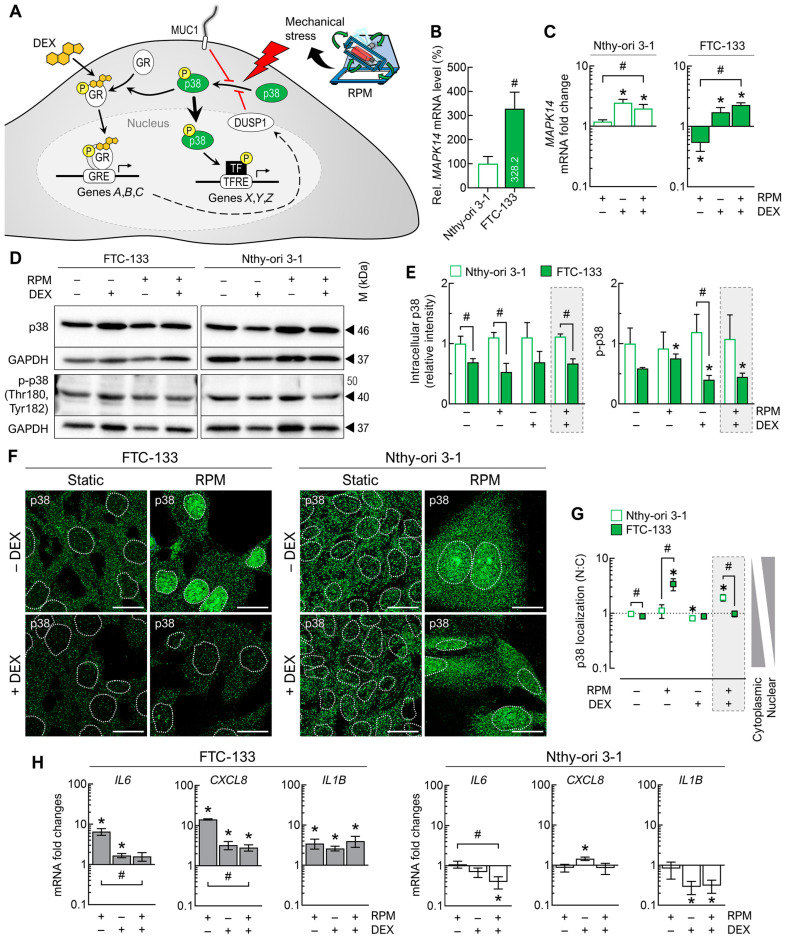
Effect of DEX on p38 MAPK and p38 signaling in FTC-133 and Nthy-ori 3-1 cells after 3 days. (**A**) Simplified schematic representation of p38′s mechanism of action. GRE: glucocorticoid response element, TF: transcription factor, TFRE: transcription factor response element. (**B**) Expression levels (mRNA) of the *MAPK14* gene (*n* = 5); (**C**) fold changes of *MAPK14* expression under the different culture conditions (*n* = 5); (**D**) intracellular protein levels of p38 and phosphorylated p38 (p-p38) indicated by Western blot (see also Supplementary File S1); (**E**) densitometric quantification of Western blot proteins (*n* = 3); (**F**) p38 immunofluorescence in adherently growing cells. p38 is shown in green, with the outlines of the nuclei as dashed lines. (**G**) Nuclear localization of p38 (*n* = 5); (**H**) fold changes in the expression of some mutual signature genes of the p38 pathway under the different culture conditions (*n* = 5). Scale bars: 300 µm. * *p* < 0.05 vs. control condition (−DEX/−RPM); ^#^ *p* < 0.05.

A crosstalk between GR and p38 mitogen-activated protein kinase (MAPK) was reported earlier [43,44,45]. p38 (gene symbol: *MAPK14*) can be activated by phosphorylation through mechanical forces [46] and then either accelerates the activation of GR or migrates to the nucleus, where it can itself affect various transcription factors and thus control the expression of numerous genes (Figure 8A). *MAPK14* was expressed about three-fold higher in the cancer cells (Figure 8B), and transcription was upregulated by DEX treatment in both FTC-133 cells and Nthy-ori 3-1 cells. However, downregulation of *MAPK14* by random positioning was also observed in FTCs (Figure 8C).

The p38 protein levels were generally higher in Nthy-ori 3-1 cells compared with FTC-133 cells and were not significantly affected by DEX treatment or the RPM. Phosphorylated p-p38 (Thr180, Tyr182), which can be transferred to the nucleus, was significantly increased in FTC-133 cells on the RPM without DEX and significantly decreased after DEX treatment. In Nthy-ori 3-1 cells, p-p38 levels were not altered by DEX or by random positioning (Figure 8D,E).

Under static cell culture conditions, p38 was mainly located in the cytoplasm of FTC-133 cells, whereas on the RPM, p38 was shifted into the nucleus. This translocation did not occur in the presence of DEX. In contrast, in Nthy-ori 3-1 cells, p38 was shifted into the nucleus only in the presence of DEX (Figure 8F). Thus, we found a strong activation of the p38 pathway during random positioning in FTC-133 cells without DEX and a slight activation of this pathway in Nthy-ori 3-1 cells with DEX (Figure 8G).

Genes under the control of p38 include *IL6*, *CXCL8* and *IL1B*. Consistent with the nuclear localization of p38, all three genes were upregulated on the RPM in FTC-133 cells, and for *IL6* and *CXCL8* we found attenuation of the mRNA levels on the RPM in the presence of DEX. In Nthy-ori 3-1 cells, gene expression was not affected by the RPM alone, but *IL6* and *IL1B* were downregulated on the RPM in the presence of DEX (Figure 8H).

## 4. Discussion

In this study, we showed that DEX is not only able to suppress RPM-induced spheroid formation of FTC-133 follicular thyroid cancer cells, but this inhibition was selective for two metastatic thyroid carcinoma cells, WRO and FTC-133, whereas benign Nthy-ori 3-1 cells and recurrent ML-1 thyroid cancer cells were not affected by DEX.

### 4.1. Effects of Dexamethasone

DEX is commonly used in clinical practice to treat side effects such as nausea and vomiting caused by chemotherapy. Wu et al. [47] pointed out that DEX itself is able to inhibit tumor cells in vitro and in vivo. That DEX may also have an anti-metastatic effect is supported by several findings. In a recent study, DEX not only decreased the viability of T47D human breast cancer cells in a time- and dose-dependent manner, but also reduced their cell adhesion and migration. This effect was explained by the altered expression of α-/β-integrin, E-/N-cadherin and MMP-2/-9 in response to DEX treatment [48]. Other authors described a disruption of dynamic cytoskeletal organization in these cells when they were treated with DEX [49]. Lin et al. [50,51] previously observed suppression of ovarian cancer metastasis by DEX. In human bladder cancer cells, suppression of MMP-2, MMP-9 and IL-6 expression, as well as induction of mesenchymal-to-epithelial transition (MET were found in the presence of DEX [52]. Therefore, the described effects of DEX on the RPM metastasis model are not entirely unfounded.

Control of GR by PTEN (phosphatase and tensin homolog) appears to be a fail-safe mechanism for tumor suppression [53]. The loss of PTEN activity in FTC-133 cells might affect GR levels, which could explain the lower translocation of GR into the nucleus of FTC-133 cells in the presence of DEX. In endothelial cells, shear stress led to nuclear localization of GR and consequent expression from the GRE promoter [54,55]. This we could not detect with our thyroid cells on the RPM. While the RPM seems to play a rather minor role in YAP1/Hippo signaling in the adherent cell population, FTC-133 cells responded to random positioning with strong activation of p38. This p38 stress signaling in FTC-133 cells could be inactivated by DEX, maybe due to the kinase activity of GR-induced DUSP1, which was upregulated after DEX treatment. In comparison, Nthy-ori 3-1 cells also activated p38 on the RPM but to a lesser extent and without sensitivity to DEX. Thus, DEX directly interferes with RPM-induced stress signaling via p38 in FTC-133 cells and antagonizes a variety of gene expressions and cell functions according to the multiple transcriptional substrates of p38 [56].

### 4.2. RPM-Induced Spheroid Formation and Its Inhibition by Dexamethasone

It has been previously reported in the literature that both healthy (Nthy-ori 3-1) and low-differentiated follicular thyroid cancer cells (ML-1, FTC-133, and WRO) form spheroids on the RPM within 24 h [57,58,59,60,61,62]. All these cells required a lag time of several hours before some of the cells detached from the bottom of the culture flask and assembled into 3D aggregates, while others remained attached. According to the latest comparison by Warnke et al. [58], no striking difference was found between healthy and cancer cells with respect to morphological changes induced by random positioning. It was therefore even more surprising that DEX specifically inhibits the spheroid formation of metastatic thyroid carcinoma cells on the RPM. This suggests that the RPM-induced spheroid formation of the different cell types may not be identical. To date, very few studies have addressed the question of how spheroid formation on the RPM is affected by drugs or the targeted blocking of functional proteins (Table 1). Therefore, it is very difficult to obtain insightful data from this works.

Some years ago, Lin and Wang [65] summarized the use of glucocorticoids in cancer treatment. In their literature review, they found different effects, in particular that glucocorticoid treatment could promote the growth of malignant solid tumors in certain cancers, while playing a suppressive role in tumor progression and metastasis in other cancers. They concluded that simple cellular experimental models are not sufficient to accurately predict therapeutic outcome in vivo [65]. According to the current literature, space science-derived RPM [66,67] enables a complex, though not yet fully understood, in vitro model for adherent cancer cell metastasis [19,20,21,68]. Since RPM also promotes 3D growth of other benign cells, it is understandable that there must be some differences between spheroid and tumor spheroid formation, although the visible result is the same in the end. Comparisons between FTC-133 and Nthy-ori 3-1 RPM cell cultures have been performed in the past. Kopp et al. [57] suggested that growth and angiogenic factors may be responsible for the differences in RPM-induced spheroid formation between malignant and healthy thyroid cells. Warnke et al. [58,59] confirmed the involvement of cytokines and focal adhesion proteins during the RPM culture of both cell lines. However, these comparisons were primarily related to gene and protein expression of markers with a focus on the response to altered gravity conditions. In this study, we took a more functional phenotypic approach to explain why benign thyroid cells respond differently to DEX on the RPM treatment than the corresponding carcinoma cells. In this way, we could determine that the adhesion of tumor and healthy thyroid cells on the RPM is affected differently.

We observed, under normal culture conditions, that DEX increased the adhesion of both cell types. This can be explained, on the one hand, by the increased expression of fibronectin (only in Nthy-ori 3-1 cells) and, on the other hand, by an increased formation of TJs, which was particularly observed in metastatic FTC-133 and WRO cells. Glucocorticoid-induced TJ formation and re-organization has been previously described and studied in mouse mammary epithelial cells [69,70] and in preclinical human intestinal models (summarized in [71]). In our experiments, the RPM also increased the expression of TJ proteins claudin-1 and ZO-1, as well as the formation of TJs, which had an enhancing effect when combined with DEX treatment. In a clinorotation experiment with MCF-7 breast cancer cells, Adamian et al. [72] recently observed similar increases in claudin-1 and claudin-3 after 72 h.

It is known that changes in the expression and/or distribution of TJ proteins can lead to a loss of cohesion of the TJ structure. This, in turn, gives cancer cells the ability to invade and ultimately metastasize [73]. In general, low expression of TJs is observed in highly metastatic cancer cells [74], and dedifferentiation of thyroid carcinomas is associated with a decrease in claudin-1, -4, and -7 expression [75]. In FTC-133 cells, our data confirmed low TJ protein expression. However, intracellular localization of claudins is also important, as pure overexpression of non-junctional nuclear claudin-1 in FTC-133 cells led to increased cell migration and invasion [76].

In addition, we also found an increase in AJs in FTC-133 when the cells were treated with DEX on the RPM. AJs have a similar structural organization to TJs: cadherins form contacts with catenins, and these complexes connect the cell surface to the cytoskeleton. In particular, decreased expression or loss of cadherins has been associated with epithelial tumor development, invasion and metastasis [77]. In addition, for the RPM metastasis model, Sahana et al. [64] found that blocking E-cadherin with an antibody increased the tumor spheroid formation of MCF-7 cells. In thyroid cancer, E-cadherin expression is considered a potential predictive factor for clinical disease progression [78]. Huang et al. [79] reported that the loss of cell adhesion in CGTH W-2 thyroid cancer cells compared with healthy thyrocytes may be due to the incomplete assembly of the cadherin–catenin complexes at the cell membrane. Thus, an increase in assembled AJs, as we have seen from the co-localization of E-cadherin and β-catenin, could also lead to enhanced cell adhesion.

Therefore, it is reasonable to speculate that the increased number of TJs and AJs, especially in FTC-133 cells, could cause a reduction in cell migration after DEX treatment on the RPM, which then leads to the inhibition of tumor spheroid formation. In addition, it would be important to clarify whether the increased occurrence of cell–cell junctions influences the detachment behavior of the cancer cells. It is possible that coherent aggregates of cells, rather than individual cells, detach. For this investigation, an RPM microscope would have to be available, which is currently not the case. Furthermore, this alone does not answer the question of why RPM-induced spheroid formation of healthy Nthy-ori 3-1 cells is not inhibited by DEX, where the amount of ECM proteins and cell junctions is as high or higher than in FTC-133 cells.

### 4.3. Spheroids from Healthy Cells on the RPM—A Question of Anti-Adhesion?

Cell surface-associated mucins such as mucin-1 are involved in the protection of epithelial surfaces from mechanical stress [80]. This protection program seems to be activated in adherent Nthy-ori 3-1 and ML-1 cells on the RPM, but not in FTC-133 and WRO cells. Because mucin-1 was upregulated in Nthy-ori 3-1 cells only in the RPM samples (not by DEX alone), a hypoxia-induced effect, which has been described in other epithelial cells for other mucins [81], can be excluded here. One possibility could be RPM-induced expression of *MUC1* via STAT3, as recently described in chondrocytes [82]. Due to steric hindrance of mucin-1 and adhesion proteins, overexpressed mucin-1 has an anti-adhesion effect on cells [83]. Intracellularly, it leads to loss of cell–cell contact and induces anchorage-independent growth including resistance to anoikis [84,85]. Both effects could favor the detachment of Nthy-ori 3-1 cells during random positioning, which show much stronger adhesion than metastatic FTC-133 cells under normal culture conditions (Figure 3D). They could even allow the formation of spheroids from cells that would otherwise not detach during random positioning, or undergo anoikis after detachment. Indeed, the absence of such an unexpected mucin-1 increase may explain why not all benign or primary human cells readily form 3D structures on the RPM. Spheroid formation on the RPM is more frequently observed in tumor cells, which tend to spread or migrate and generally have higher mucin expression [86]. One of the best-known example pairs studied so far on the RPM is probably MCF-7 mammary tumor cells and MCF-10A mammary epithelial cells. Monti et al. [86] showed that only MCF-7 cells formed spheroids on the RPM after 72 h. In addition, we found by immunofluorescence analysis that MCF-10A cells expressed very little mucin-1 protein (Figure S2B). Triple-negative MDA-MB-231 cells, which also have very low levels of mucin-1 [87], tend to adhere to culture flasks on the RPM rather than detach (unpublished data). It is also known that *MUC1* gene expression in prostate cancer cells is inhibited by the androgen receptor (AR) [88]. In some of our preliminary experiments, AR-positive LnCAP cells detached less from the growth surface after administration of dihydrotestosterone. Mucin-1 immunofluorescence staining confirmed a decrease in protein amount in the presence of dihydrotestosterone (Figure S2C). In addition, RPM-induced spheroid formation was observed to be absent in some primary endothelial cells. While human umbilical vein endothelial cells (HUVECs) are able to form 3D aggregates, this does not work for human saphenous vein endothelial cells (HSVECs) under RPM conditions [89]. At least for HUVECs, it was demonstrated that mucin-1 is expressed and can be released from the cell membrane [90]. However, we could not find any information about mucin-1 in HSVECs so far.

In this study, we attempted to target mucin-1 pharmaceutically. Although GO-203 is primarily an inhibitor of MUC1-C and thus blocks its intracellular signaling, an auto-inductive effect that has been described for mucin-1 [91] could explain the reduction of (surface) mucin-1 observed after supplementation of GO-203 (Figure 6G).

In summary, it seems important to note that not only adhesion proteins play a role in RPM-induced spheroid formation, but also anti-adhesion proteins.

## 5. Conclusions

For gravitational biologists, the RPM offers a good platform to study tumor progression or metastasis in vitro and to uncover special targets for cancer therapy [19,20,21,68]. In this study, we could show that RPM-grown thyroid cancer cells behave differently than corresponding benign epithelial cells when treated with DEX. Comparative analyses showed that the cell detachment process (which is often referred to as ‘in vitro metastasis’) during random positioning is selectively suppressed by DEX in metastatic cancer cells; on the one hand, due to massive formation of TJs in these cells, and, on the other hand, due to the upregulation of anti-adhesive mucin-1 in their non-metastasis-derived relatives by the RPM. The overall picture of an initially hypothesized anti-metastasis effect of DEX in vitro emerged from an interplay of different effects of the cells, all of which responded differently to RPM cell culture. The balance between adhesion and anti-adhesion appears to allow detachment of adherent human cells on an RPM. This depends on the origin of the cells, their development, and their consequent susceptibility to stress. Therefore, it will continue to be very important to study exactly how human cells behave in rotating bioreactors in order to derive the maximum benefit from these model systems for translational medicine.

## Figures and Tables

**Table 1:** Other studies on targeting RPM-induced spheroid formation of cancer cells.

Cell Line	Drug	Main Findings	Reference
FTC-133	Dexamethasone	Inhibition of spheroid formation.	[31]
MCF-7	Dexamethasone	Reduced spheroid formation.	[63]
	Rolipram	No effects.	
	Olaparib	No effects.	
MCF-7	PP2	Inhibition of spheroid formation.	[64]
	anti-E-cadherin	Increased spheroid formation.	

## Data Availability

The data presented in this study are available in the electronic Supplementary Material. The complete raw data are available on request.

## Supplementary Materials

The following supporting information can be downloaded at: https://www.mdpi.com/article/10.3390/cancers15061641/s1, Figure S1: Effect of DEX (1000 nM) and random positioning on tight junction proteins after 3 days; Figure S2: Mucin-1 expression in relation to cell adhesion of different cell lines in RPM experiments; Figure S3: Oxygen saturation and hypoxia-induced expression of *HIF1A* in completely filled cell culture flasks; Table S1: Antibodies used for immunofluorescence and Western blot analyses; Table S2: Primer sequences for quantitative real-time PCR. File S1: Full-size Western blots.
