# Supplementary material for: Dexamethasone Selectively Inhibits Detachment of Metastatic Thyroid Cancer Cells during Random Positioning
Source: Cancers (Basel). 2023 Mar 7;15(6):1641. doi: 10.3390/cancers15061641 (PMC10046141; doi:10.3390/cancers15061641)

FTC-133 – MMP2

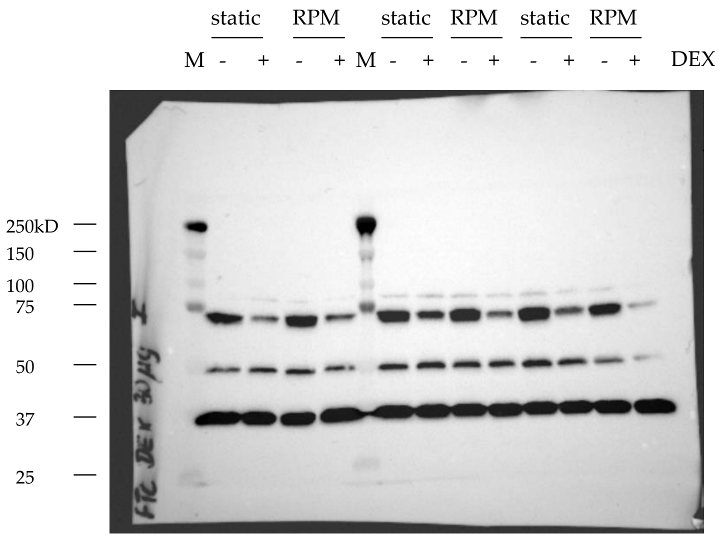

FTC-133 – GAPDH to MMP2

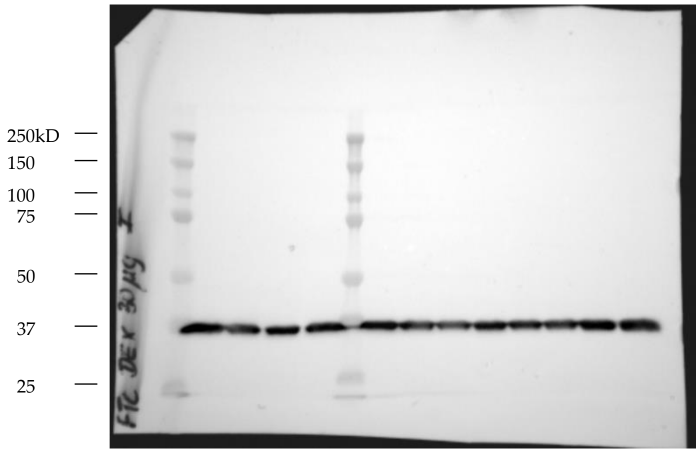

FTC-133 – MMP9

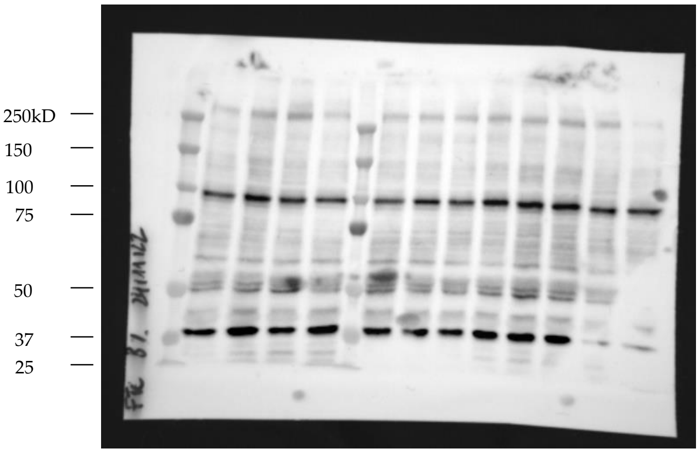

FTC-133 – GAPDH to MMP9

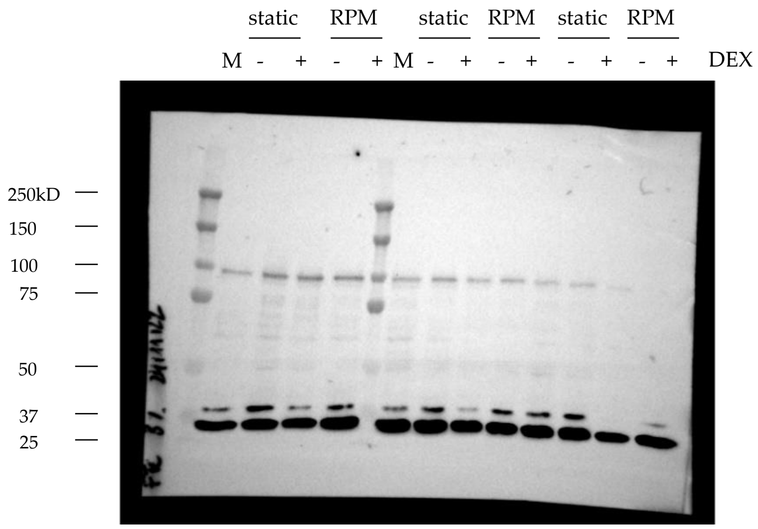

FTC-133 – pGR

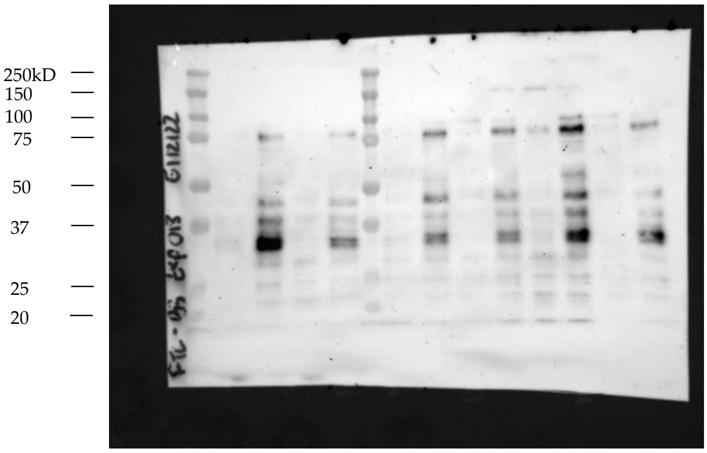

FTC-133 – GAPDH to pGR

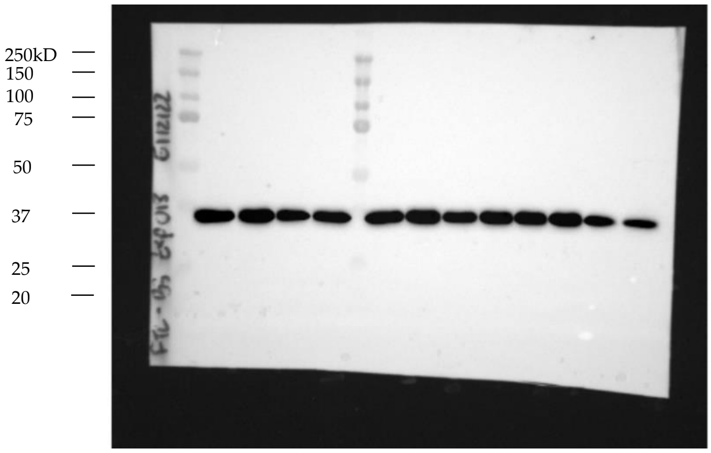

FTC-133 – GR

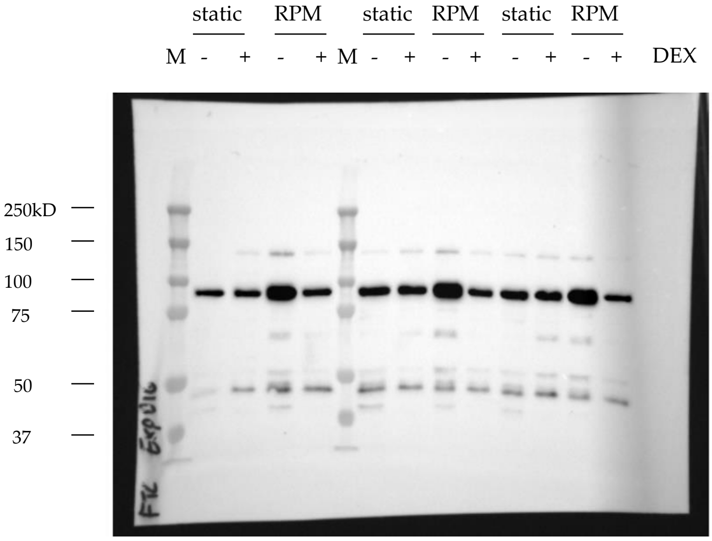

FTC-133 – GAPDH to GR

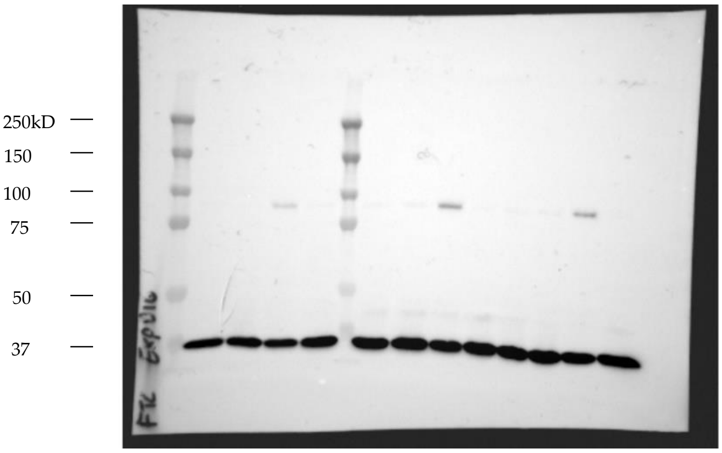

FTC-133 – p38

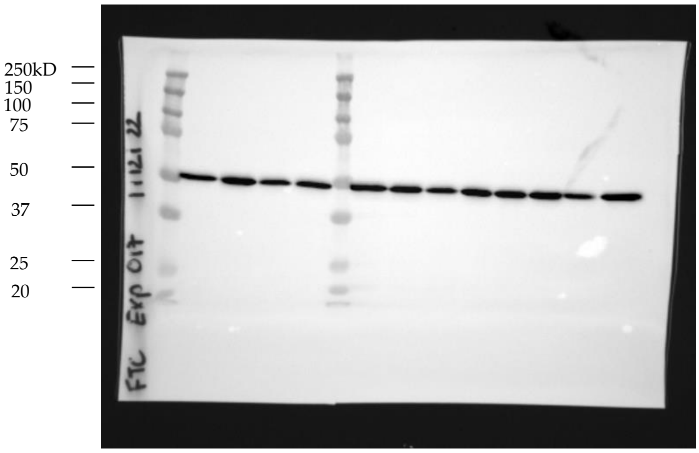

FTC-133 – GAPDH zu p38

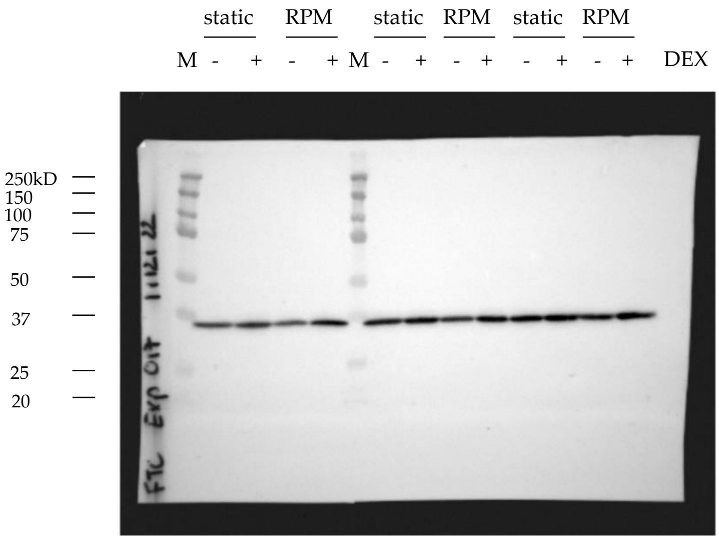

FTC-133 – pp38

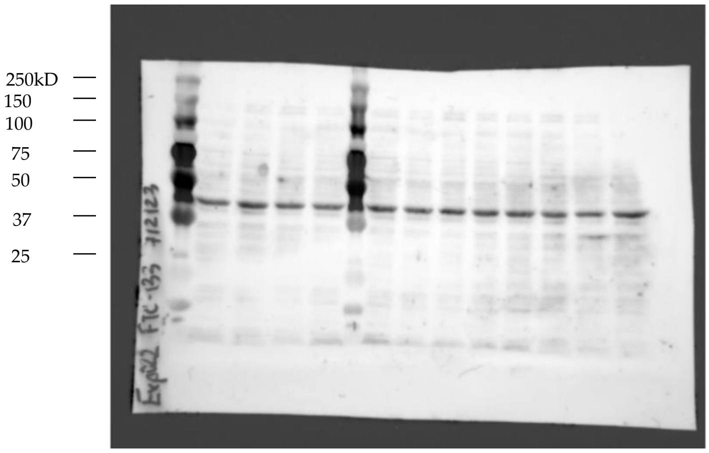

FTC-133 – GAPDH zu pp38

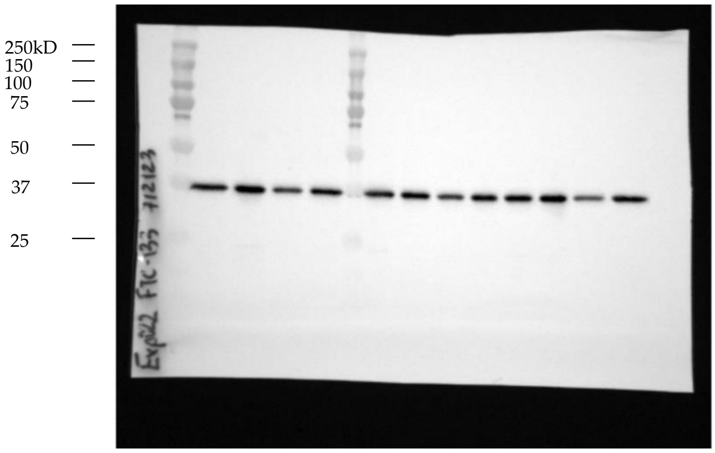

Nthy-ori 3-1 – GR

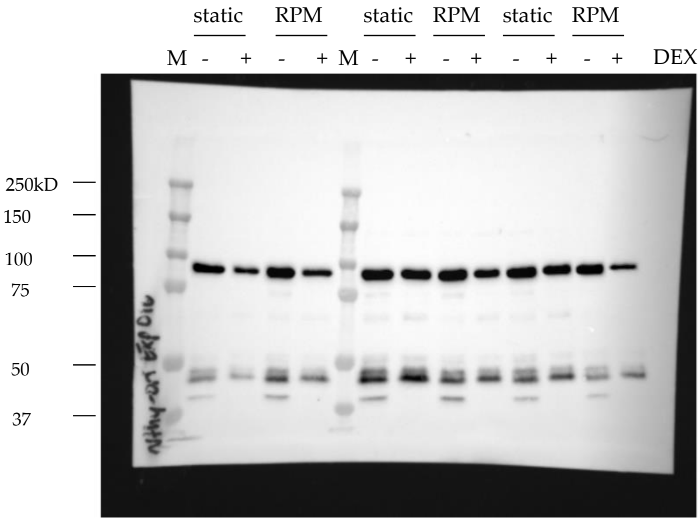

Nthy-ori 3-1 – GAPDH to GR

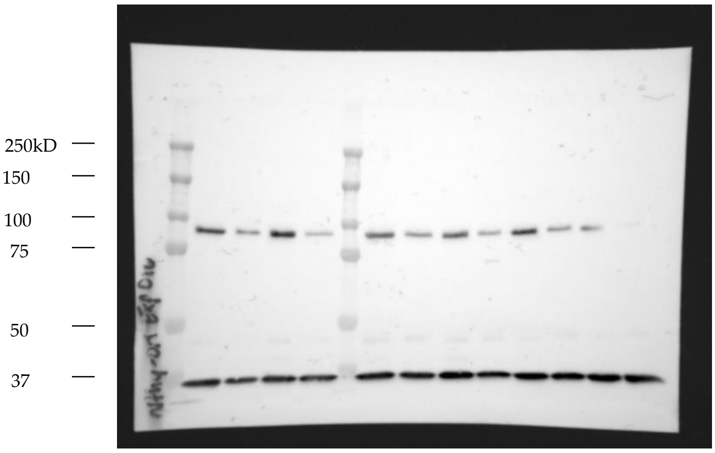

Nthy-ori 3-1 – p38

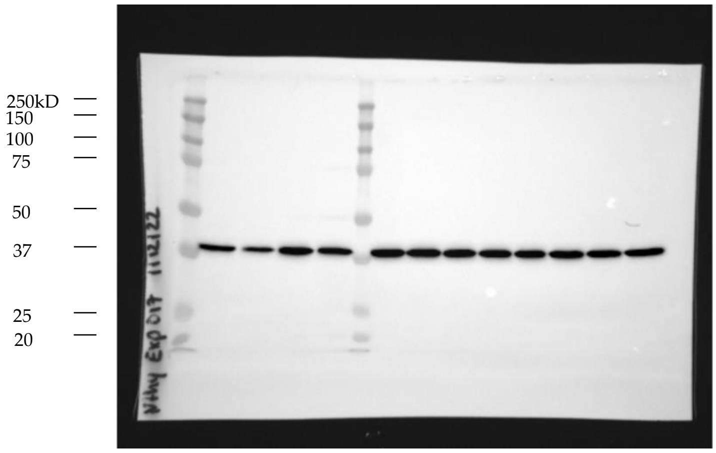

Nthy-ori 3-1 – GAPDH to p38

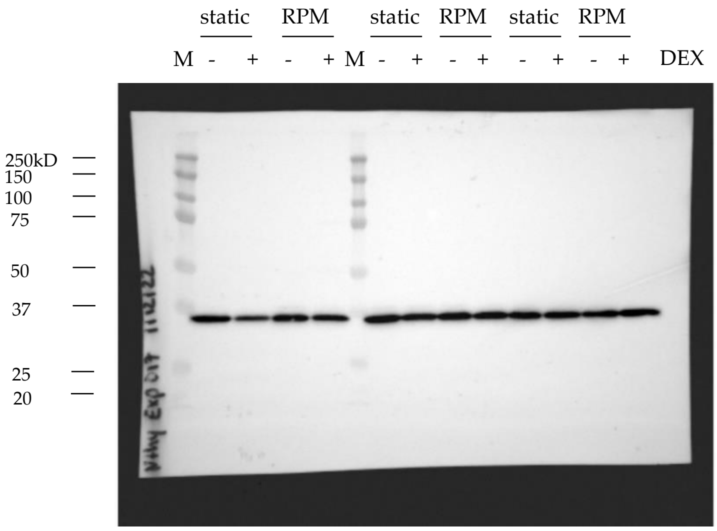

Nthy-ori 3-1 – MMP9

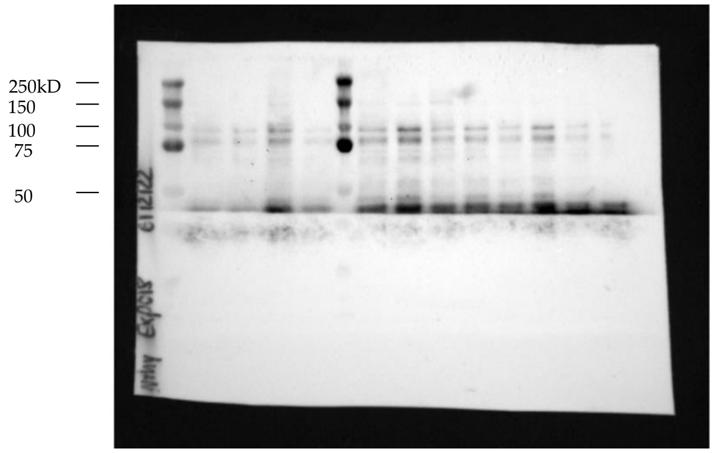

Nthy-ori 3-1 – pGR

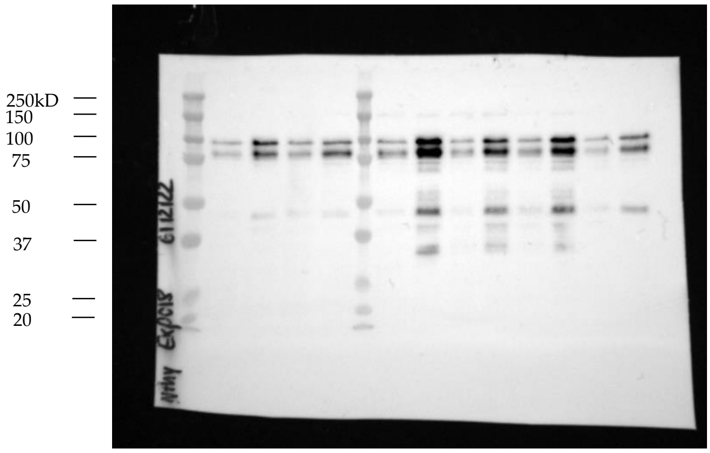

Nthy-ori 3-1 – GAPDH to MMP9 and pGR

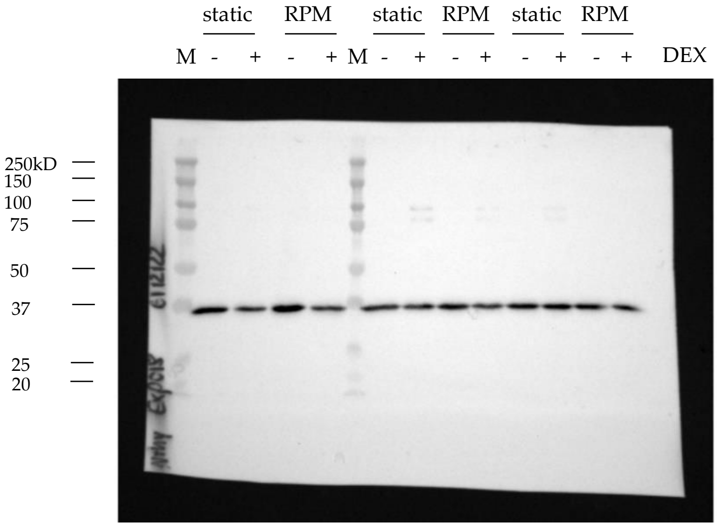

Nthy-ori 3-1 – pp38

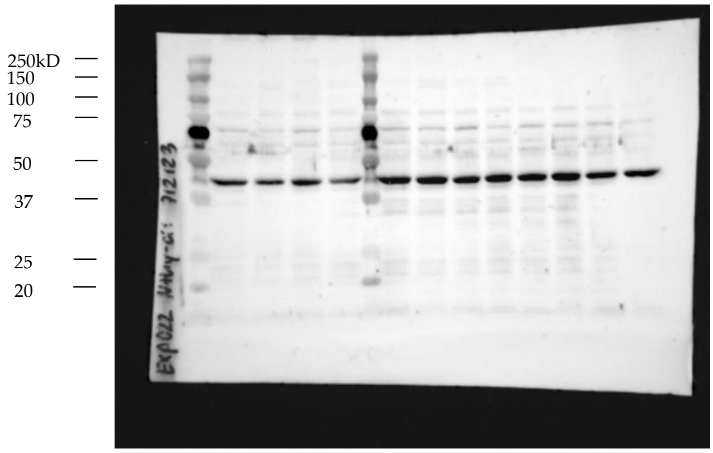

Nthy-ori 3-1 – GAPDH to pp38

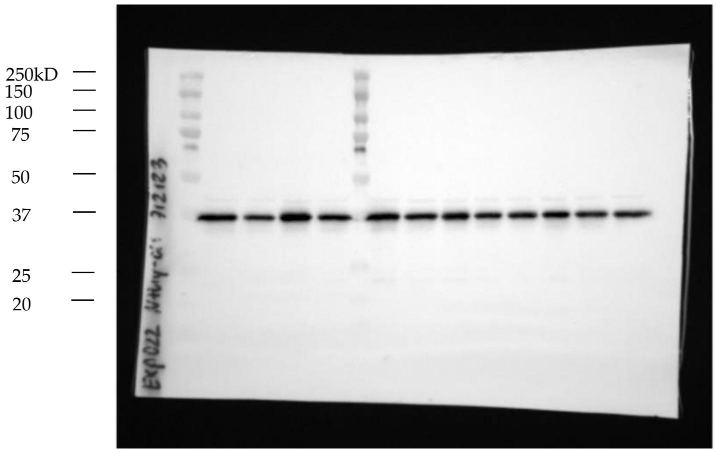

Nthy-ori 3-1 – MMP2

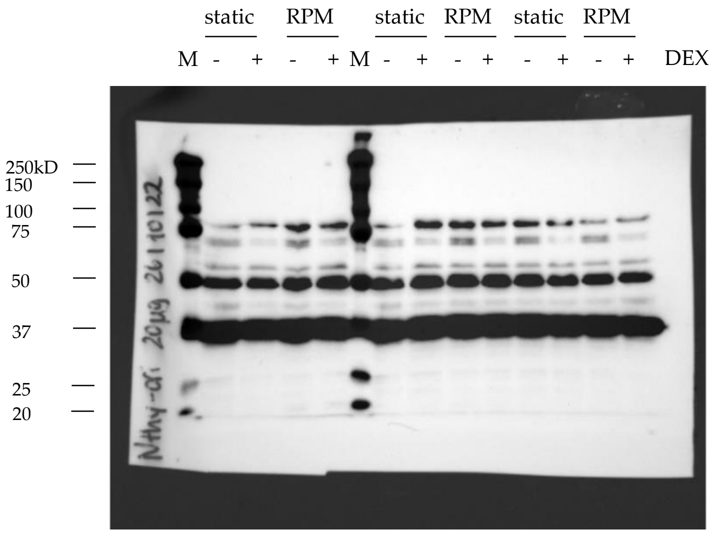

Nthy-ori 3-1 – GAPDH to MMP2

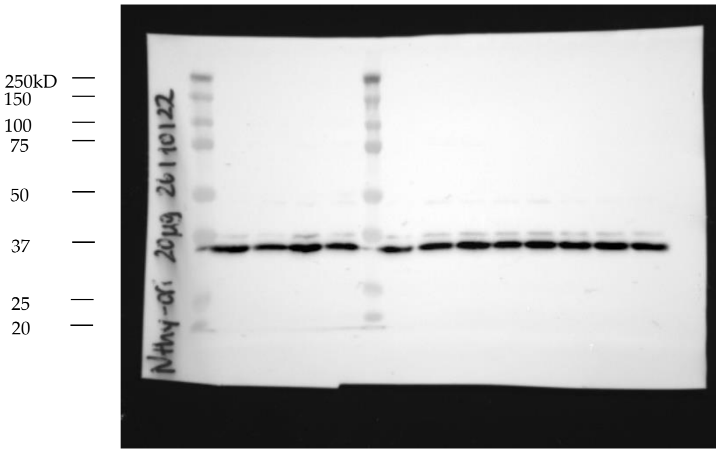

Supplement: Supplementary file 1 [file cancers-15-01641-s001.zip › cancers-2247443-supplementary File S1.pdf]
