# Supplementary material for: Dexamethasone Selectively Inhibits Detachment of Metastatic Thyroid Cancer Cells during Random Positioning
Source: Cancers (Basel). 2023 Mar 7;15(6):1641. doi: 10.3390/cancers15061641 (PMC10046141; doi:10.3390/cancers15061641)
Supplement: Supplementary file 1 [file cancers-15-01641-s001.zip › cancers-2247443-supplementary.pdf]

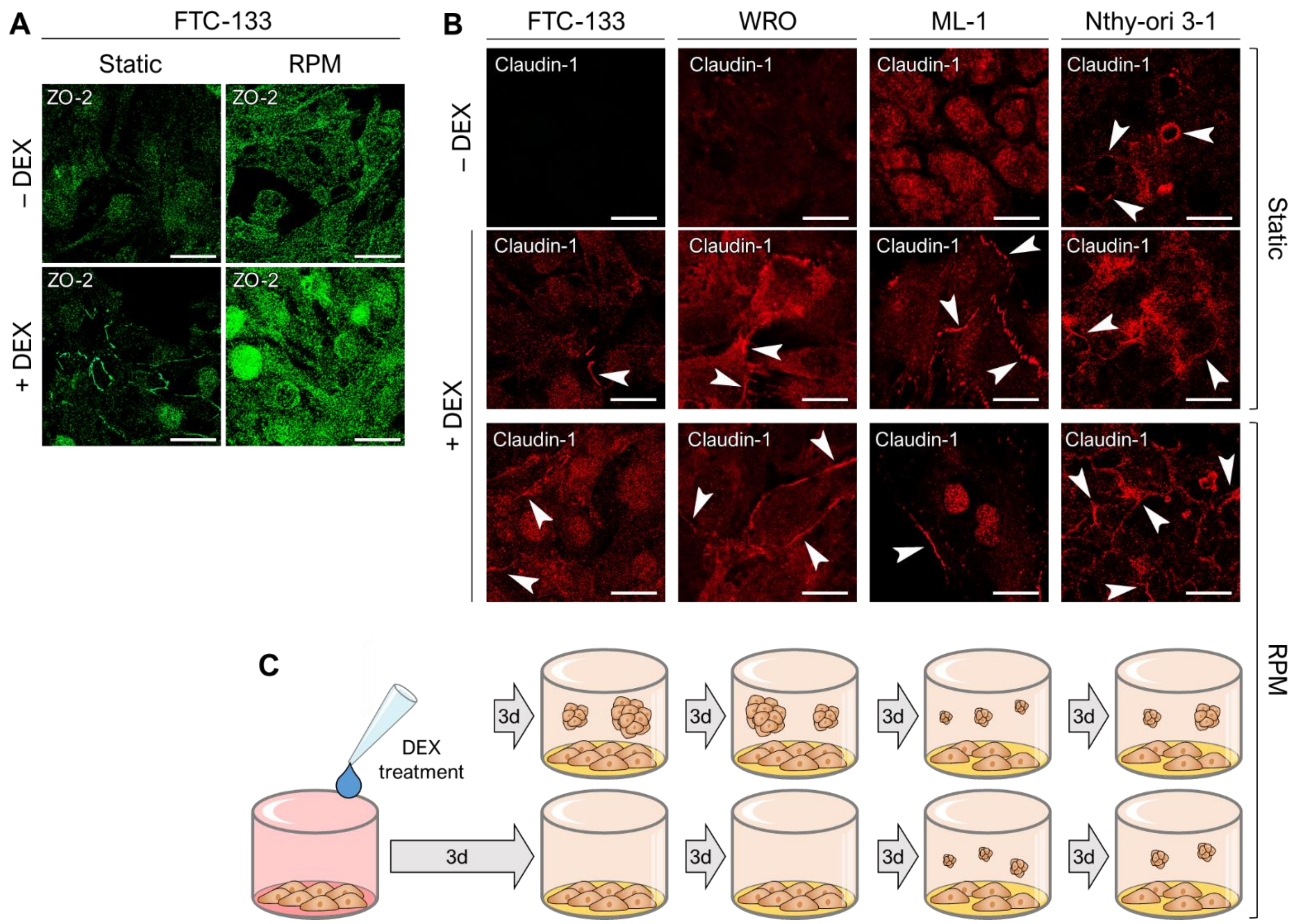

Figure S1. Effect of DEX (1000 nM) and random positioning on tight junction proteins after 3 days. (**A**) Immunofluorescence of ZO-2 (green) under the different culture conditions; (**B**) Immunofluorescence of claudin-1 (red) in different cell under static and RPM conditions. Arrows indicate the accumulation of claudin-1 at cell borders for the formation of tight junctions; (**C**) Schematic illustration of spheroid formation in the culture conditions shown in subfigure (B). Scale bars: 300 µm.

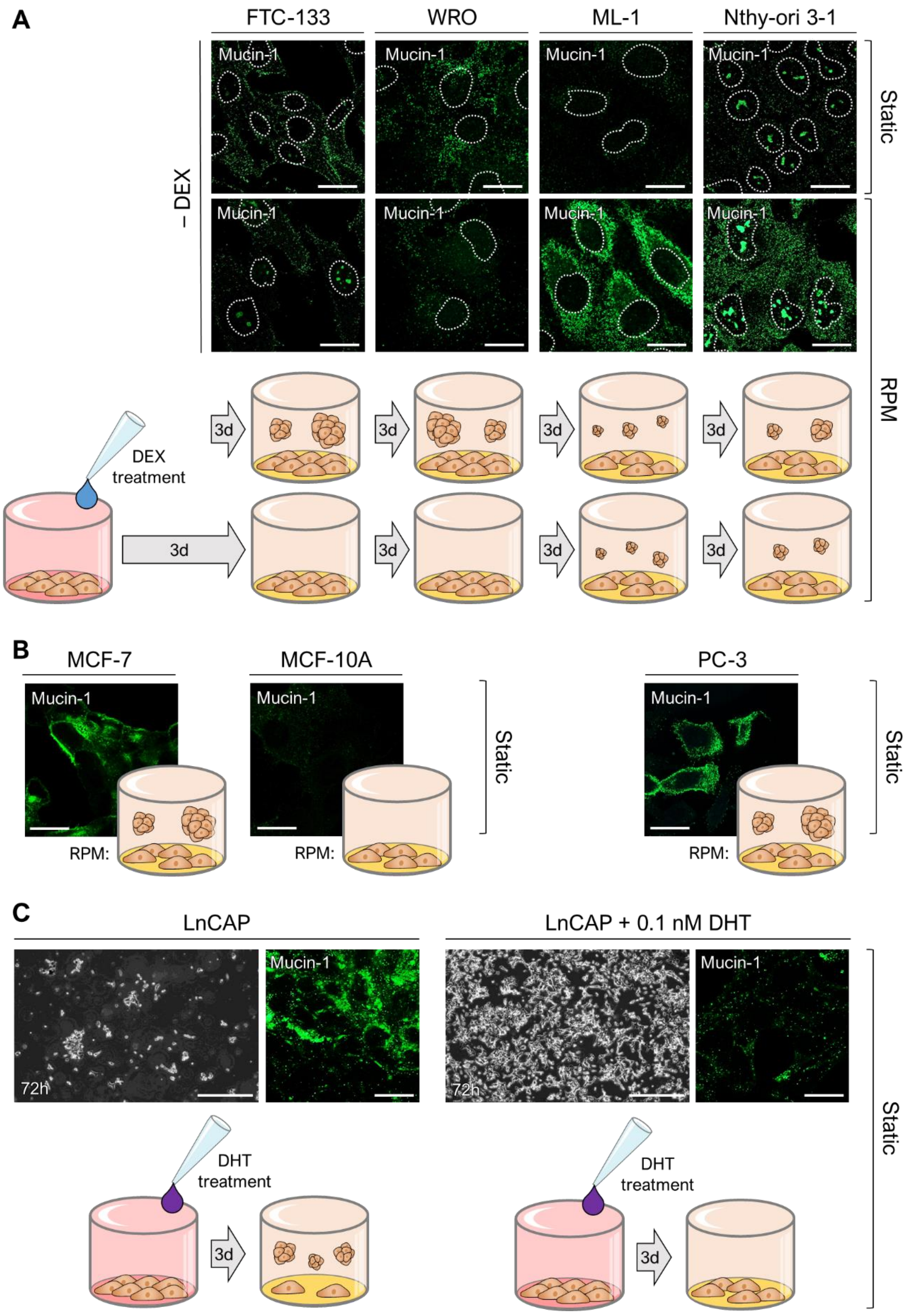

Figure S2. Mucin-1 expression in relation to cell adhesion of different cell lines in RPM experiments. (**A**) Immunofluorescence of RPM-induced mucin-1 (green) in adherently growing thyroid (cancer) cells after 72 h. Schematic diagrams show expected spheroid formation in the respective cell cultures; (**B**) Basal mucin-1 expression (green) in other cancer cells studied on the RPM. Schematic diagrams show expected spheroid formation in the respective cell cultures; (**C**) While LnCAP prostate cancer cells in normal RPMI-1640 medium express mucin-1 (green fluorescence) on the RPM and adhere poorly, the addition of 0.1 nM dihydrotestosterone (DHT) led to a decrease in the amount of mucin-1 and thus increased adherence. Scale bars: 300 µm.

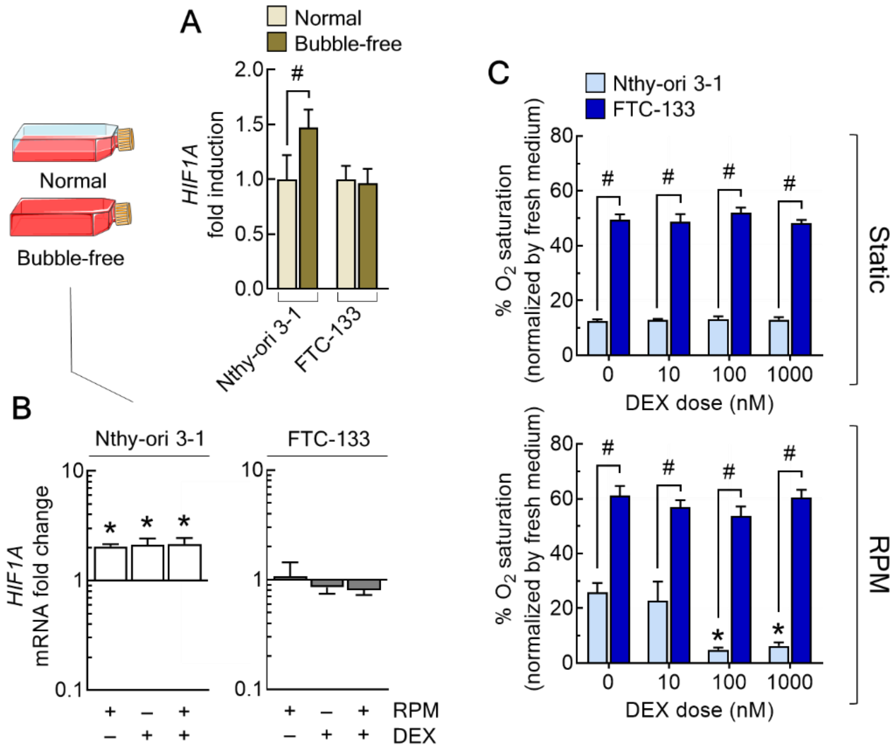

Figure S3. Oxygen saturation and hypoxia-induced expression of *HIF1A* in completely filled cell culture flasks. (**A**) Expression levels (mRNA) of the *HIF1A* gene (n=5); (**B**) Fold changes of *HIF1A* expression under the different culture conditions (n=5); (**C**) Oxygen levels in completely filled cell culture flasks after 72 h (n=3). * $p < 0.05$ vs. control condition (-DEX/-RPM); $^{\#}$ $p < 0.05$.

Table S1. Antibodies used for immunofluorescence and Western blot analyses.

**Antibody** (anti-…)	**Company, Product Nr.**	**Species**[1]	**MW** (kDa)	**Dilution IF**[2]	**Dilution WB**[2]
β-catenin	Invitrogen, #71-2700	Rb	94	3 µg/mL	
Claudin-1	Invitrogen, #37-4900	Ms		3 µg/mL	
Collagen I	Invitrogen, #MA1-26771	Ms		1:2000	
Collagen IV	Invitrogen, #PA5-104508	Rb		1:300	
E-cadherin	Invitrogen, #MA5-11496	Ms		2 µg/mL	
FAK	Abcam, #ab40794	Rb		1:1000	
Fibronectin	Sigma, #F3648	Rb		1:400	
GAPDH	Invitrogen, #398600	Ms	~37		1:2000
GR	Sigma, #HPA004248	Rb	~110	1 µg/mL	1:2000
Integrin-β1	R&D Systems, #MAB17781	Ms		1:500	25 µg/mL
Laminin	Invitrogen, #PA1-16730	Rb		1:100	
MMP-2	Invitrogen, #436000	Ms	72		2 µg/mL
MMP-9	Invitrogen, #MA5-32705	Ms	~100		1:500
Mucin-1	Invitrogen, #MA1-35039	Ms		1:100	
p38 MAPKα	Invitrogen, #MA5-15116	Ms	46	1:1000	1:1000
p-GR (Ser 211)	Invitrogen, #PA5-17668	Rb	~86		1:500
p-p38 (Thr180, Tyr182)	Invitrogen, #44-684G	Rb	~38		1:500
ZO-1	Invitrogen, #40-2200	Rb		3.5 µg/mL	
ZO-2	Invitrogen, #71-1400	Rb		1 µg/mL	

**Secondary Antibody**	**Company, Product Nr.**	**Labelling**[3]	**Dilution IF**[2]	**Dilution WB**[2]	
				Prim. AB	Factor
anti-Mouse	Invitrogen, #A-11001	AF488	1 µg/mL		
	Invitrogen, #A-21235	AF647	2 µg/mL		
	Invitrogen, #31430	HRP		p38 MAPKα	1:6000
				GAPDH	1:50,000
				MMP-2	1:2000
anti-Rabbit	Invitrogen, #A-11008	AF488	4 µg/mL		
	Invitrogen, #A-21244	AF647	4 µg/mL		
	Invitrogen, #31460	HRP		MMP-9	1:1000
				GR	1:10,000
				p-GR	1:10,000
				p-p38	1:10,000

[1] Ms: mouse; Rb: rabbit; [2] IF, immunofluorescence; WB, Western blot; [3] AF, Alexa Fluor; HRP, horseradish peroxidase.

Table S2. Primer sequences for quantitative real-time PCR.

Gene	Primer Name	Sequence (5′->3′)
18S rRNA	18S-F	GGAGCCTGCGGCTTAATTT
	18S-R	CAACTAAGAACGGCCATGCA
CXCL8	CXCL8-F	TGGCAGCCTTCCTGATTTCT
	CXCL8-R	GGGTGGAAAGGTTTGGAGTATG
DUSP1	DUSP1-F	AGGACAACCACAAGGCAGAC
	DUSP1-R	TCCAGCATTCTTGATGGAGTCTATG
FKBP5	FKBP5-F	AACCCCCAGAATAAGGCTGC
	FKBP5-R	TTGGCGTATATCCTGCGGTC
HIF1A	HIF1A-F	TGCTTTAACTTTGCTGGCCC
	HIF1A-R	AGTTTCTGTGTCGTTGCTGC
IL1B	IL1B-F	TTCGAGGCACAAGGCACAA
	IL1B-R	TGGCTGCTTCAGACACTTGAG
IL6	IL6-F	CGGGAACGAAAGAGAAGCTCTA
	IL6-R	GAGCAGCCCCAGGGAGAA
MAPK14	MAPK14-F	TGTTTCCTGGTACAGACCATATT
	MAPK14-R	CATGGCTTGGCATCCTGTT
MUC1	MUC1-F	CCTCACAGTGCTTACAGTTGTT
	MUC1-R	GCTGGGCACTGAACTTCTCT
MYC	MYC-F	GGATTCTCTGCTCTCCTCGAC
	MYC-R	CTTCTTGTTCCTCCTCAGAGTC
NR3C1	NR3C1-F	GTTGTTTATCTCGGCTGCGG
	NR3C1-R	TCAGTGAATATCAACTCTGGCA
